# Robust pose estimation which guarantees positive depths

**DOI:** 10.1038/s41598-023-49553-9

**Published:** 2023-12-13

**Authors:** Chun Li, John E. McInroy

**Affiliations:** 1 Department of Electrical and Computer Engineering, University of Wyoming, Laramie, Wyoming 82071 USA; 2 Beijing Synchrotron Radiation Facility, Institute of High Energy Physics, Chinese Academy of Sciences, Beijing, 100049 China; 3 Spallation Neutron Source Science Center, Dongguan, 523803 Guangdong China

**Keywords:** Electrical and electronic engineering, Mathematics and computing

## Abstract

In the area of 3D computer vision, the ability to estimate pose between two cameras under high noise levels while maintaining small reprojection errors reflects the robustness of such pose estimation algorithms. Moreover, maintaining positive depth constraint is another challenging task. Unfortunately, current pose estimation algorithms are often sensitive to noise/outliers and do not always guarantee positive depths. As a standalone task, these algorithms perform a positive sign check and simply discard the points with negative depths after the algorithms are executed. These algorithms do not integrate positive depth constraints into the algorithms themselves. Instead, they do it afterwards. Here, from a comprehensive mathematical derivation, we propose a novel pose estimation algorithm that integrates positive depth constraint into the algorithm itself by estimating the depths directly. The algorithm was competitive in producing small reprojection errors when compared to the state-of-the-art algorithms under both synthetic and real-world tests, while most importantly guaranteeing positive depths.

## Introduction

Pose estimation is widely and thoroughly studied in the field of computer vision, which tackles the problem of solving relative pose between cameras or world coordinate systems. It is proven useful in various real-world computer vision application scenarios like Structure from Motion (SfM)^1^, Simultaneous Localization and Mapping (SLAM)^2^, Light Detection and Ranging (LIDAR)^3,4^, autonomous navigation^5,6^, Augmented Reality (AR)^7,8^, and so on. From a model-wise perspective, the input of pose estimation problem is camera observations, usually denoted as uncalibrated pixel coordinates or calibrated image coordinates. The output is pose parameters between coordinate systems, specifically, rotation matrix, translation vector, depth, etc. Most importantly, the integral part that connects the input and the output is known as pose estimation algorithm. In literature, pose estimation is often interchangeably used with terms such as Perspective-n-Point (PnP). Therefore, PnP algorithms are also considered pose estimation algorithms, which comprise both 3D-2D and 2D-2D PnP algorithms according to different nature of observations.

Over the decades, pose estimation algorithms or PnP algorithms have evolved dramatically, on both algorithmic design and performance. According to the nature of the observations, state-of-the-art (SOTA) algorithms can be categorized into 2D-2D and 3D-2D methods, which require intrinsic camera information for pose estimation. Representative 2D-2D algorithms include Eight-point (8-pt)^9^, normalized Eight-point (8-pt (norm))^10^, Minimum-eigenvalue (Min-eig)^11^, Five-point (5-pt)^12,13^ algorithm. These 2D-2D algorithms accept 2D-2D calibrated image coordinates as inputs. On the other hand, popular 3D-2D algorithms include EPnP^14^, EPnP-GN^15^, DLS^16^, RPnP^17^, LHM^18^, DLT^19^, OPnP^20^, MLPnP^21,22^, and (R)EPPnP^23^ algorithms, and also more recently, the SQPnP^24^, CPnP^25^, Uncertain-PnP or PnP(L)^26^, and QPEPs^27^. These solvers usually try to achieve certain optimization goals. For example, due to the linearity nature, 8-pt and 8-pt (norm) focus on execution efficiency, which is simple and fast; 5-pt algorithm only requires five correspondence pairs to recover pose, extending its compatibility in more scenarios; EPnP algorithm demonstrates competitive performance with low computational cost; RPnP algorithm further improves the performance over EPnP with similar computational cost, excelling in translation estimates; OPnP performs well for all variables including rotation, translation, and depth, even under presence of noise; (R)EPPnP incorporates outlier rejection scheme into the algorithm itself, circumventing the time-consuming Random Sample Consensus (RANSAC)^28–32^ procedure, which is frequently utilized to remove outliers from the input dataset; and finally, MLPnP is a maximum likelihood solution to the PnP problem that includes image observation uncertainties to improve accuracy. Such uncertainties do occur in real-world scenarios since not all points are measured with equal certainty. For example, imperfections on sensors could lead to such uncertainties, resulting shifts on pixels. To an extent, MLPnP can also handle outliers, with outlier scrutinization process also embedded inside the algorithm. The more recent SQPnP treats PnP problem as a non-linear quadratic program (NLQP), and always determines the global minima of the PnP problem “for any number of input correspondences”, with tolerance on coplanar configurations^24^. The CPnP algorithm is another recent progress that introduces consistent estimator for the camera pose via a bias-eliminated closed-form solution, which later is refined by the constrained Gauss–Newton (GN) iterations^25^. The techniques used to integrate these two algorithms into our work for comparison can be found in Methods.

Despite the distinctive merits of the above algorithms, these SOTA algorithms encounter common yet demanding challenges, mainly on the need of noise tolerance and positive depth guarantee (positive depth constraint). On the one hand, being able to tolerate high noise levels shows the algorithms’ robustness and potential of real-world adoption; on the other hand, since physically depth signs must all be positive (points are in the front of camera), being capable of guaranteeing positive depth estimates eliminates the need of cheirality check^33^ (sign check) after algorithm execution, which is a redundant, time-consuming yet unavoidable task that requires multiple steps of manual intervention. Such intervention consists of removing extraneous solutions containing negative depth estimates, or even requires elimination of certain data corresponding to negative depths, which causes data loss. Unfortunately, current SOTA algorithms can hardly maintain robustness, while also conforming to positive depth constraint. For instance, the highly robust OPnP algorithm has no guarantee that the depth estimates will be all positive. Therefore, being able to be robust to noise while simultaneously guaranteeing positive depth is a critical concern for current pose estimation algorithms.

Here, through complete mathematical derivation, a pose estimation algorithm called Pos-dep (or Min-eig-Depths) is proposed. The algorithm is specifically designed to guarantee positive depth estimates while demonstrating robustness to noise changes, yielding small reprojection errors compared to the SOTA. The cheirality check process that examines the signs of the depths is integrated into the proposed algorithm itself, circumventing extra checks on the signs. Based on a solid mathematical backbone, the algorithm iteratively solves the rotation, and the eigenvector of a data matrix that contains all positive entries as the depth vector. The proposed algorithm obtains positive depth estimates even before the pose is estimated, making cheirality check and solution selection no longer necessary by providing a unique solution. In addition, the algorithm is robust and adapts to noisy conditions in terms of increasing noise intensities and presence of outliers, showing its robustness to noise changes.

## Results

We will first present the results obtained using simulated data. Then, we present the results obtained in real-world scenarios. We separated the experiment on synthetic data (simulation) into three subsections: varying the noise levels and varying the percentage of outliers to fully test on the algorithms' robustness to noise and outliers, and varying the number of points to test the performance and computation time when more points are added.

### Varying the noise levels

We performed the experiments on the range of standard deviation (std) of zero-mean Gaussian noise covering from 1e − 7 to 1e − 1 in calibrated image coordinates. To cover such wide noise spectrum, we used “semilog” plots for the *x*-axis. The *y*-axis was the percentage of good estimates so that the higher the plot, the better the performance. The number of iterations done at each noise level was 100. The number of points was ten.

**Figure 1:**
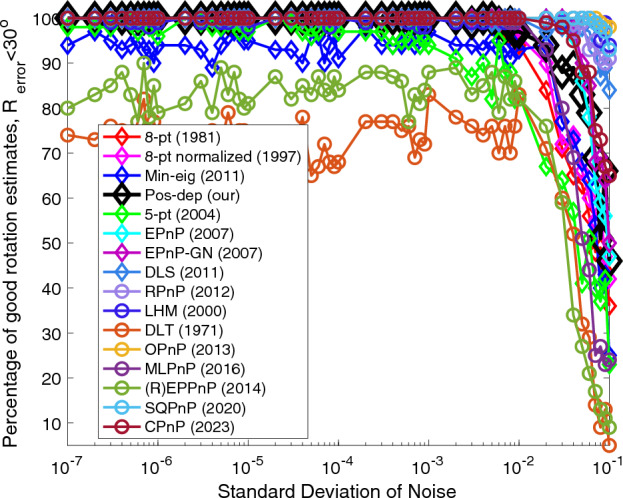
Rotation (< 30◦) vs. noise levels.

**Figure 2:**
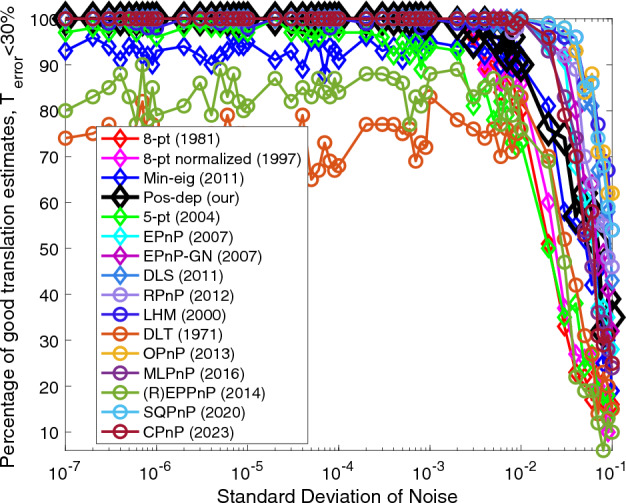
Translation (< 30%) vs. noise levels.

**Figure 3:**
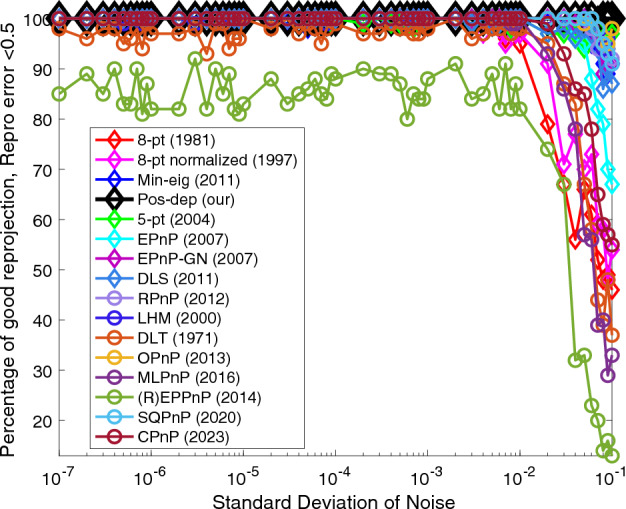
Reprojection (< 0.5) vs. noise levels.

**Figure 4:**
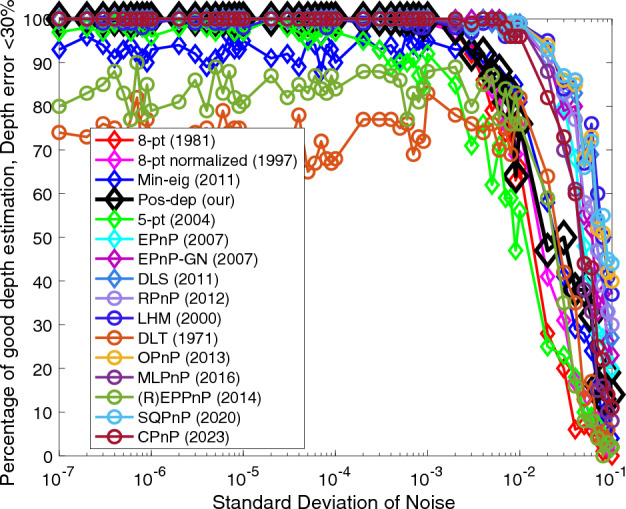
Depth (< 30%) vs. noise levels.

Figures 1, 2, 3 and 4 show all algorithms’ percentages of good rotation, translation, reprojection, and depth estimates under varying noise levels. By saying “good”, we are referring that the estimation errors are less than some set thresholds. It can be seen from Fig. 1 that the Pos-dep was among the best ones, it only downgraded after the noise std was beyond 1e − 2. The DLT and (R)EPPnP were the worst. We can also see that our Pos-dep algorithm was the best among all 2D-2D algorithms, keeping at a percentage of 100 when the noise was below 1e − 2. After that, it also had the slowest degrading rate compared to other 2D-2D algorithms. The SQPnP outperformed CPnP when the noise std exceeds 1e − 2.

For translations, we see from Fig. 2 that the Pos-dep stayed at 100% line until 3e − 3, after that it kept comparable with 3D-2D algorithms such as MLPnP and EPnP. Among all 2D-2D algorithms, our Pos-dep beat all others when the noise was below 3e − 3; when the noise was beyond this level, our algorithm still remained comparable to the Min-eig, while beating others, including the 8-pt (norm). The SQPnP outperformed CPnP when the noise std exceeds 1e − 2.

From Fig. 3, we see for reprojections, of all the algorithms the Pos-dep performed the best (stayed up at the 100% line), including the 3D-2D PnP algorithms and the recent SQPnP and CPnP. In fact, being capable of reaching and maintaining small reprojection errors across the noise spectrum is one of the main advantages of the Pos-dep algorithm. The SQPnP outperformed CPnP when the noise std exceeds 2e − 2 (0.02).

For depth estimates, we see from Fig. 4 that overall, the 3D-2D algorithms were better than the 2D-2D, with DLT and (R)EPPnP being the exceptions. Among all 2D-2D algorithms, our Pos-dep again beat all other 2D-2D algorithms, especially at low-medium noise levels. The SQPnP outperformed CPnP when the noise std exceeds 1e − 2.

**Figure 5:**
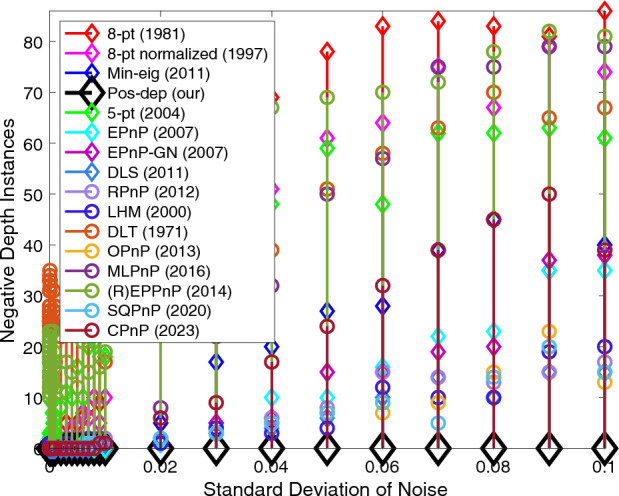
Negative depth instances vs. noise levels.

In terms of negative depth instances, we can clearly see from Fig. 5 that out of 100 tests on each noise level, the Pos-dep stayed all the way flat at the zero-instance line, guaranteeing positive depth estimates. The SQPnP algorithm overall has fewer instances of negative depths than the CPnP algorithm.

**Figure 6:**
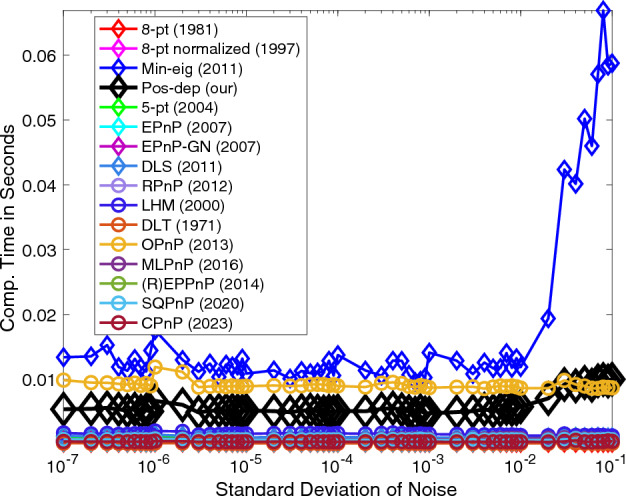
Computation time vs. noise levels.

The computation time of Pos-dep was consistently around 6 ms across the noise spectrum, lower than the OPnP algorithm which was around 10 ms (Fig. 6). The runtime was recorded using MATLAB on a PC with 16 GB RAM and 6-core Intel CPU with 2.6 GHz clock frequency.

Limited by the length of this paper, for more comprehensive results that involve testing with smaller thresholds and comparison within the 3D-2D and 2D-2D algorithmic pools only, please refer to pages 194–210 of this thesis^34^.

**Figure 7:**
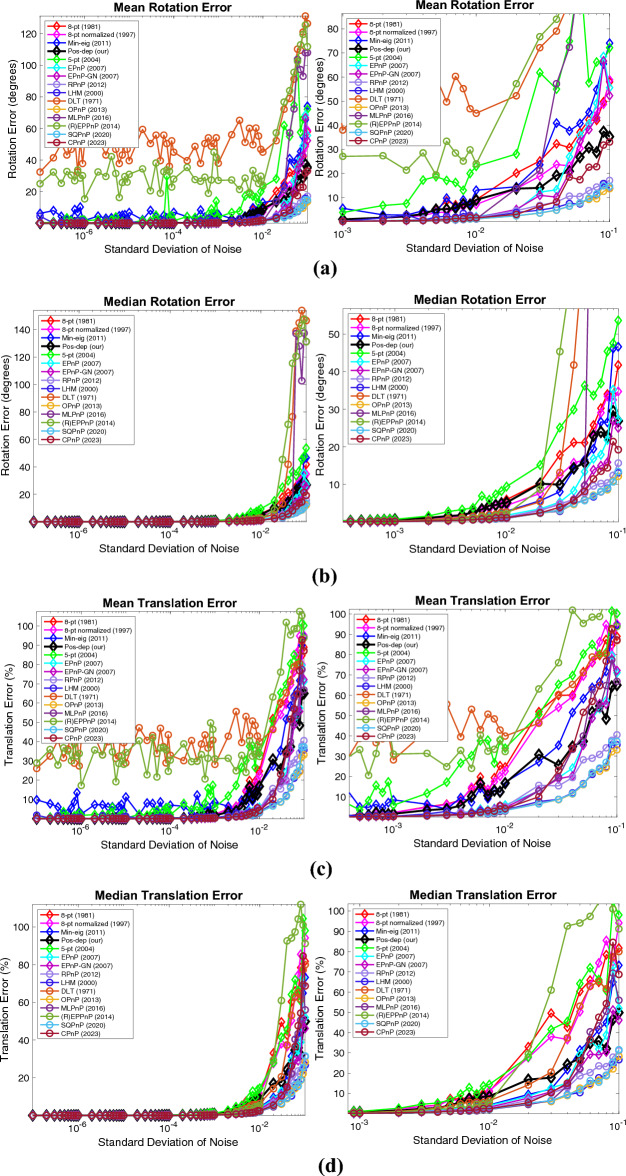

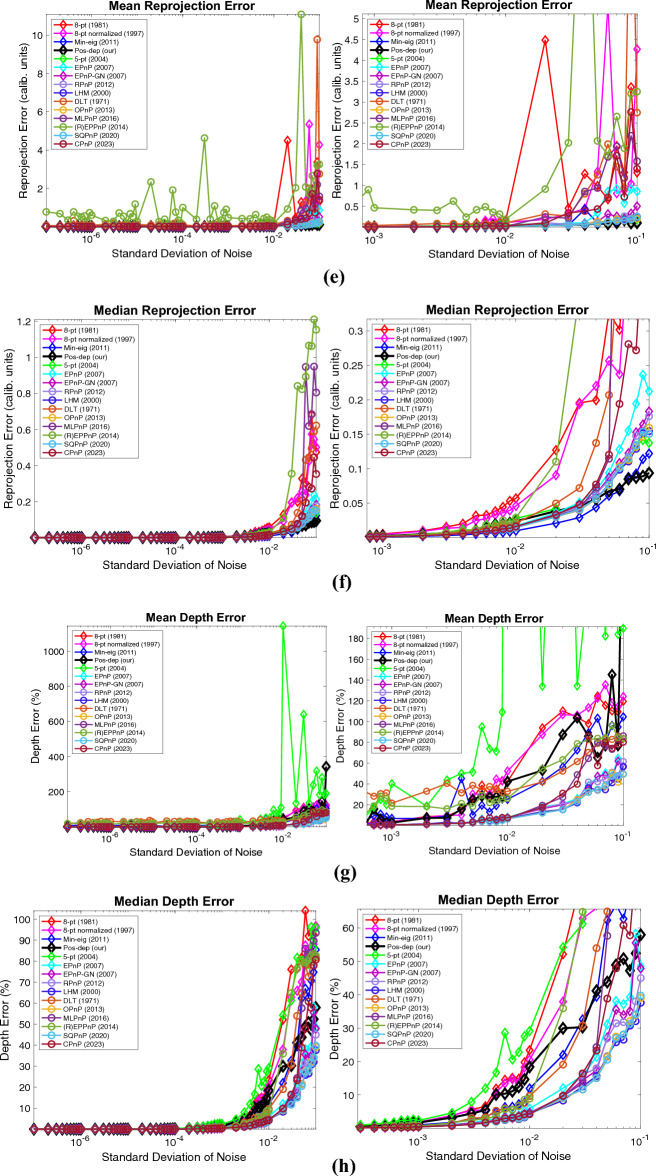
Mean and median estimation errors when varying the noise levels. (**a**) Mean rotation error, a close-up is shown to the right; (**b**) Median rotation error, a close-up is shown to the right; (**c**) Mean translation error, a close-up is shown to the right; (**d**) Median translation error, a close-up is shown to the right; (**e**) Mean reprojection error, a close-up is shown to the right; (**f**) Median reprojection error, a close-up is shown to the right; (**g**) Mean depth error, a close-up is shown to the right; (**h**) Median depth error, a close-up is shown to the right.

In addition to the percentage of good estimates, mean and median estimation errors are also the metrics actively used in this area, as is performed in the OPnP paper^20^. Here, at each noise level, 100 runs again are independently executed. The mean and median error at each noise level is calculated. The DLS algorithm is excluded in the plot since it is out of bounds. Close-up plots are shown next to the originals. These results are shown in Fig. 7, which includes the results of mean and median rotation, translation, reprojection, and depth errors.

For rotations, in Fig. 7a and b, when the noise std is at 1e − 2, the median rotation error of Pos-dep was around 5 degrees. For translations, in Fig. 7c and d, when the noise std is at 1e − 2, the median translation error of Pos-dep was around 10%. For reprojections, we can see from Fig. 7e and f that both the mean and median reprojection errors of Pos-dep were among the smallest, compared with other algorithms. For depths, we can see from Fig. 7g and h that when the noise std is below 1e − 2, the depth errors were below 30%, which was used as the threshold to differentiate “good” estimates. From Fig. 7, we also found the OPnP and the SQPnP had similar performance, and overall performed the best in the algorithm pool.

### Varying the percentage of outliers

We define the outliers to be the data points corrupted by noise with a std of 0.1 (1e-1). We chose the percentages to be from 10 to 100% with a 10% increment. The total number of points (inliers and outliers combined) was 20. Again, 100 tests were independently performed at each outlier rate. While a proportion of the points were assigned as outliers, other points were not corrupted by noise and were still considered “clean data”.

**Figure 8:**
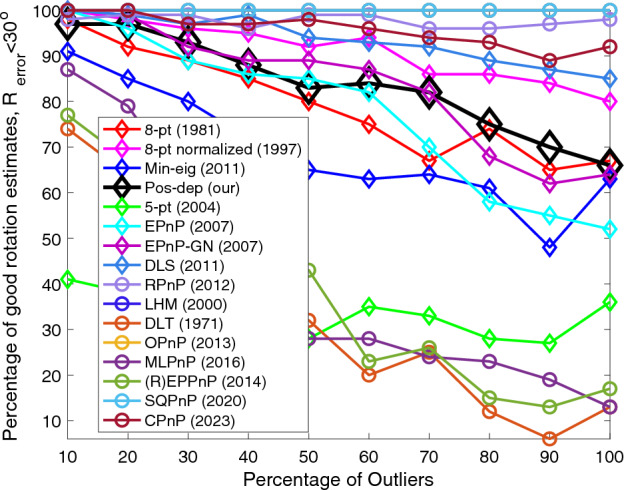
Rotation (< 30◦) vs. outlier rate.

We followed the similar evaluation method used in varying the noise levels. We can see from Fig. 8 that the Pos-dep was comparable to the EPnP-GN and better than EPnP. The OPnP and the SQPnP stayed at 100% and overlapped with each other, both were better than the CPnP algorithm. At high outlier rates such as 70%, the Pos-dep still achieved more than 80 good rotation estimates, out of 100.

**Figure 9:**
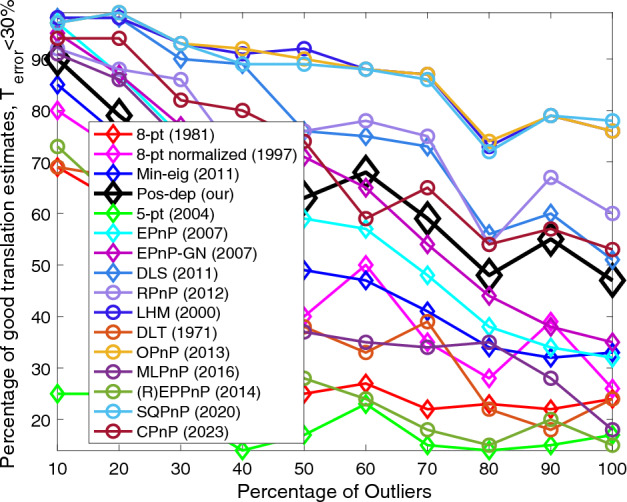
Translation (< 30%) vs. outlier rate.

Figure 9 shows that our Pos-dep again was the best among all 2D-2D algorithms. The LHM, OPnP, and SQPnP had similar performance. The SQPnP outperformed CPnP. Overall, the Pos-dep was comparable to EPnP-GN. At 60% outlier rate, the Pos-dep outperformed CPnP algorithm. At high outlier rates such as 90% and 100%, the Pos-dep can also compete with the CPnP algorithm.

**Figure 10:**
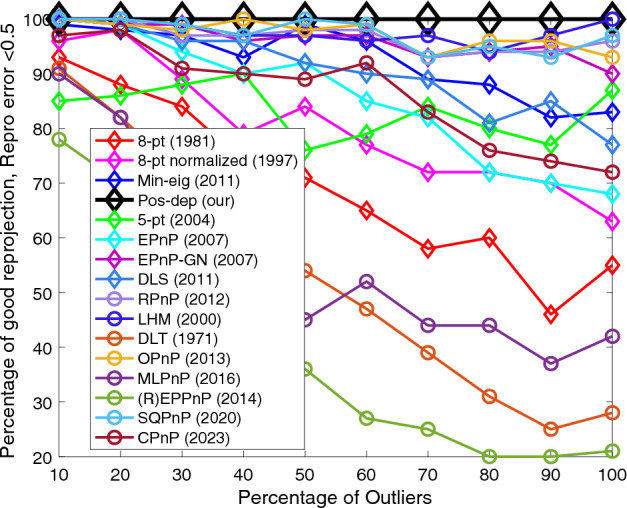
Reprojection (< 0.5) vs. outlier rate.

Figure 10 shows that for reprojections, our Pos-dep performed exceptionally well among the algorithms. It stayed at 100%. OPnP, LHM, RPnP, and SQPnP were the next. The SQPnP outperformed CPnP. The threshold used to classify good reprojection was 0.5, measured in calibrated image coordinates.

**Figure 11:**
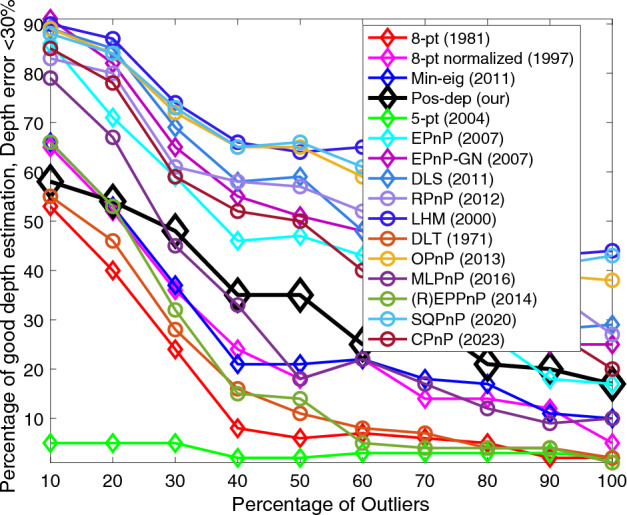
Depth (< 30%) vs. outlier rate.

Figure 11 shows that for depth errors the Pos-dep again beat all 2D-2D algorithms. The LHM, OPnP, and SQPnP were the best among all algorithms. The SQPnP outperformed the CPnP here. The threshold was 30%. Our Pos-dep was the only 2D-2D algorithm that could be comparable with the 3D-2D algorithms. In fact, it beat MLPnP when the outlier rate was beyond 30%. From Fig. 11, it can be seen our algorithm almost formed a “separating line” between the 3D-2D and 2D-2D algorithms, following the EPnP algorithm.

**Figure 12:**
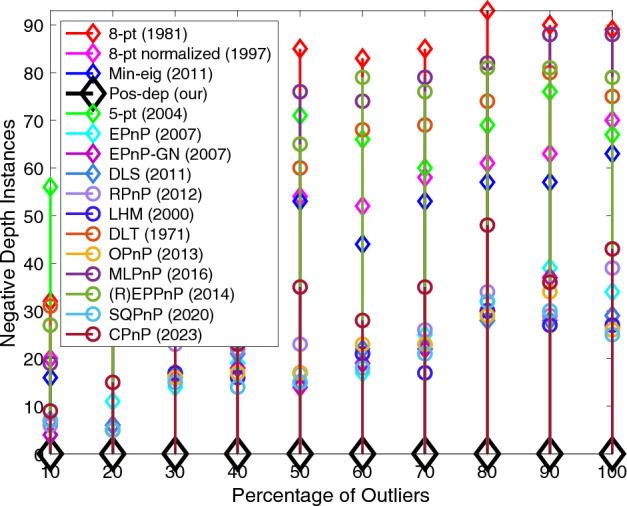
Negative depth instances.

For negative depth instances, we see from Fig. 12 that the Pos-dep stayed at the bottom of the graph all the time, meaning it recorded zero instances of negative depths. We can also see that interestingly, the negative depth instances gradually increased as the outlier rate increased. At 100% outlier rate, roughly at least 30 instances of negative depths were reported for the SOTA algorithms. The SQPnP algorithm overall has fewer instances of negative depths than the CPnP algorithm.

**Figure 13:**
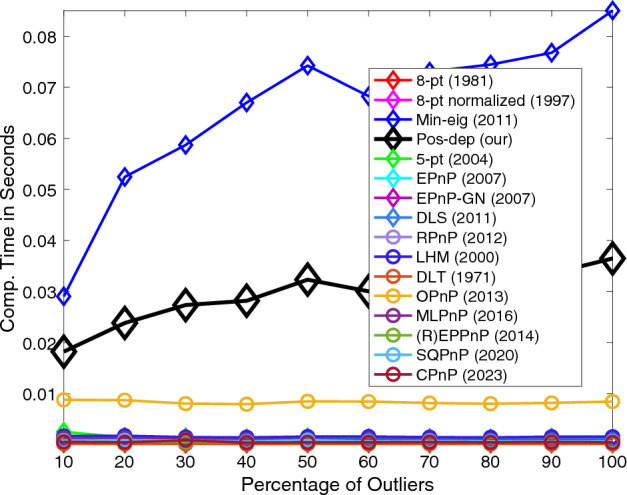
Computation time vs. outlier rate.

In terms of computation time, see Fig. 13. In theory, the computation time of Pos-dep should be nearly constant. Although there exists a slight increase of the computation time of Pos-dep, since the number of points were fixed at 20 and the computation cost of Pos-dep only depends on point numbers, we argue such increase was within the margin of reasonable system errors. For instance, as experiments going on, the machine might have gone through possible thermal throttling with reduced clock frequency that impacted the CPU’s performance. The computation time of Pos-dep ranges approximately from 20 to 40 ms, the difference is 20 ms. The OPnP stayed nearly consistently at 10 ms.

**Figure 14:**
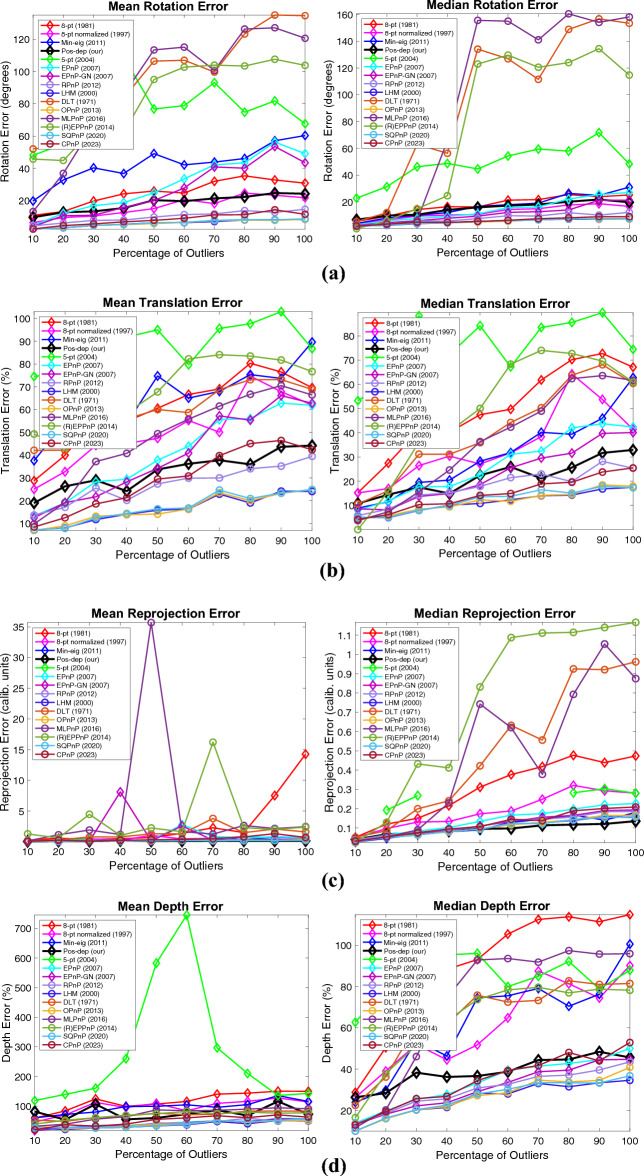
Mean and median estimation errors when varying the percentage of outliers. (**a**) Mean and median rotation errors; (**b**) Mean and median translation errors; (**c**) Mean and median reprojection errors; (**d**) Mean and median depth errors.

Similar to varying the noise levels, we also evaluated the performance by studying the mean and median estimation errors including the rotation, translation, reprojection, and depths. Again, at each outlier rate (percentage), 100 runs are independently executed. The mean and median errors at each outlier rate are calculated. The configurations such as the noise std, the choices of percentages, and the number of total points are the same as those presented in Figs. 8, 9, 10, 11, 12 and 13. The DLS algorithm is excluded since it is out of bounds. The results are shown in Fig. 14.

From Fig. 14a, we see that for rotations, the mean error of Pos-dep was comparable to that of EPnP-GN, while the median error was among the best that stayed below 20% for all outlier rates. For translations, in Fig. 14b, when the outlier rate is below 40%, the mean translation error is below 30%, and the median translation error is below 20%. In extreme circumstances such as 90% outlier rate, the mean rotation, median rotation, mean translation, and median translation errors of Pos-dep were around 20 degrees, 20 degrees, 40%, and 30%, respectively. For reprojections, from Fig. 14c we see the Pos-dep outperformed other algorithms across the outlier rates in both mean and median errors. At 100% outlier rate, the median error of Pos-dep was around 0.1, smaller than all other algorithms. For depth estimates, from Fig. 14d we see the mean errors of Pos-dep were among the best, and the median errors were the best among all 2D-2D algorithms and beat (R)EPPnP. When the outlier rate exceeds 50%, the median depth errors of Pos-dep were comparable to the most recent CPnP algorithm, although admittedly, the recent algorithms SQPnP and CPnP were among the best ones in each subplot of Fig. 14. In this experiment, the noise std used was 0.1 in calibrated coordinates, which could comfortably transform the raw data into outliers.

### Varying the number of points

**Figure 15:**
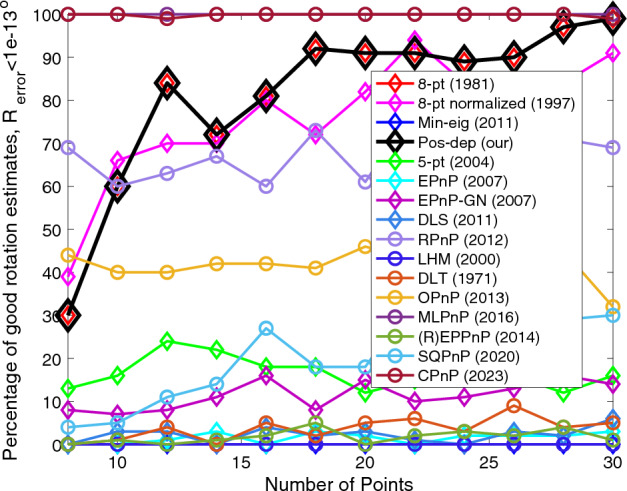
Rotation errors vs. number of points.

**Figure 16:**
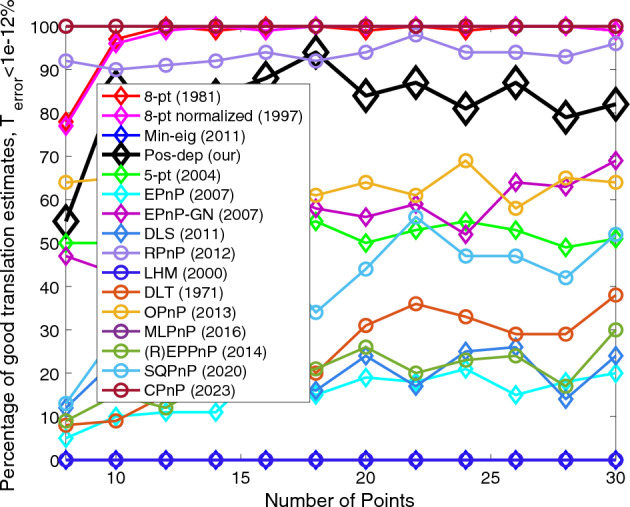
Translation errors vs. number of points.

**Figure 17:**
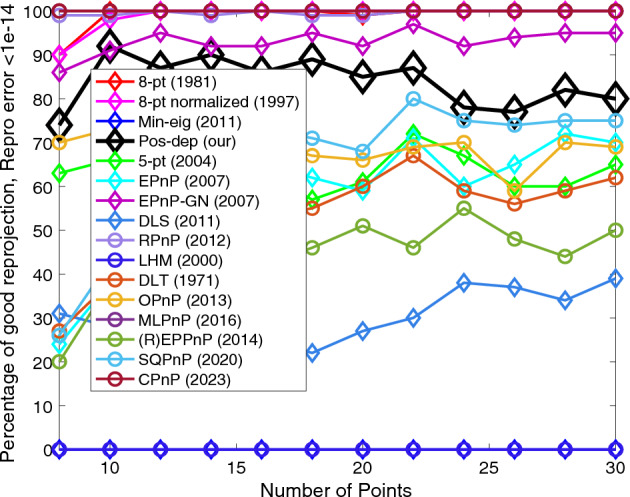
Reprojection errors vs. number of points.

**Figure 18:**
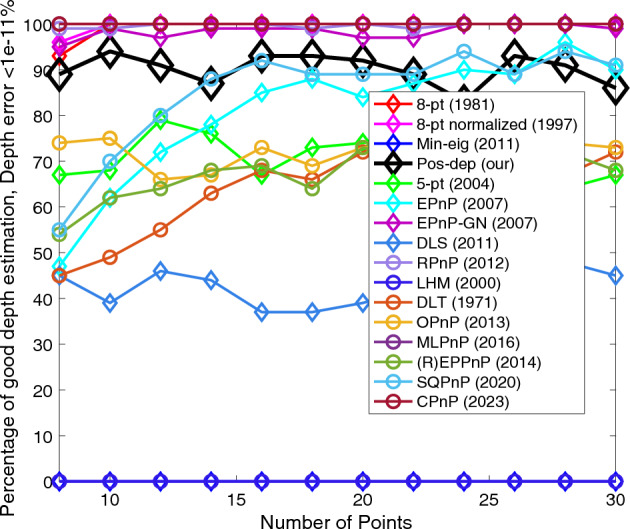
Depth errors vs. number of points.

The 8-pt algorithm requires the minimum number of points used is 8. Hence, we chose the number of points to be from 8 to 30, with an increment of 2. Figures 15, 16, 17 and 18 show the errors of rotation, translation, reprojection, and depth, respectively.

We can see from Fig. 15 that when the number of points exceeds 16, the proposed algorithm performed well (the higher the better), and the CPnP performed even better; the SQPnP only reported around 20% of good estimates. For translations we can see from Fig. 16 that the Pos-dep was only behind RPnP, which stayed above 90% in good estimates for all point numbers. The SQPnP barely reached 50%. For reprojection, we see from Fig. 17 that the Pos-dep again stayed high and was only behind EPnP-GN, which maintained above 90% in good estimates when point number was greater than 10. For depth estimates, from Fig. 18 we see the Pos-dep was better than the SQPnP algorithm, except only at point numbers 24, 28, and 30. The proposed algorithm stayed around 90% in good estimates across all point numbers.

**Figure 19:**
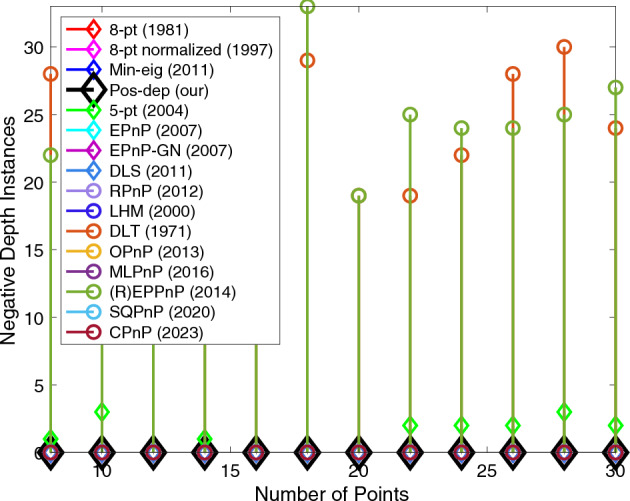
Negative depth instances vs. number of points.

The proposed algorithm reported zero instance of negative depths all the time (Fig. 19), while on average (R)EPPnP and DLT reported more than 20 negative instances out of a total of 100 when point number exceeds 18, more than other algorithms.

**Figure 20:**
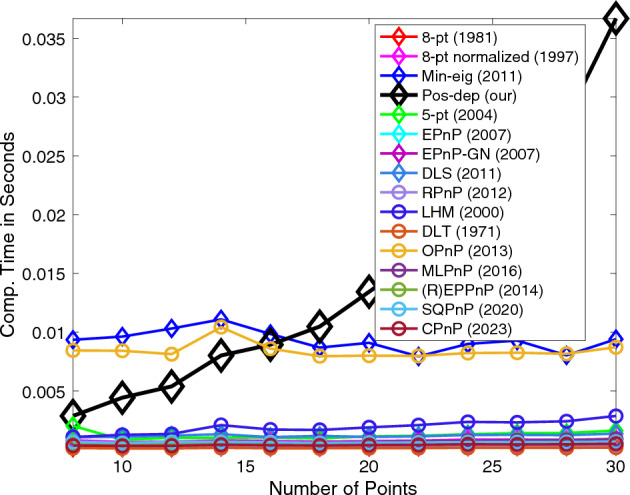
Computation time vs. number of points.

Figure 20 shows the computation time (runtime) of the algorithms versus the number of points. We see that the runtime of our algorithm almost “linearly” increased. This is expected due to the mathematical nature of our algorithm’s ${{\varvec{D}}}_{{\varvec{R}}}$ matrix, please see Eq. (23) in Methods for definition. The ${{\varvec{D}}}_{{\varvec{R}}}$ matrix is of dimension $2n\times 2n$, where $n$ is the number of points. As the number of points increase, the dimension of ${{\varvec{D}}}_{{\varvec{R}}}$ would also increase linearly, resulting in an increase of computation time when calculating eigenvectors and eigenvalues of the matrix. It is noted that even when the number of points is 30, the proposed algorithm still reported a runtime below 40 ms. For each point number, 100 runs were independently performed at each point number and the averaged runtime over these runs was reported for each point number. The reader is also encouraged to refer to pages 212 to 215 of the thesis^34^ for more comprehensive results when varying the number of correspondence points.

**Figure 21:**
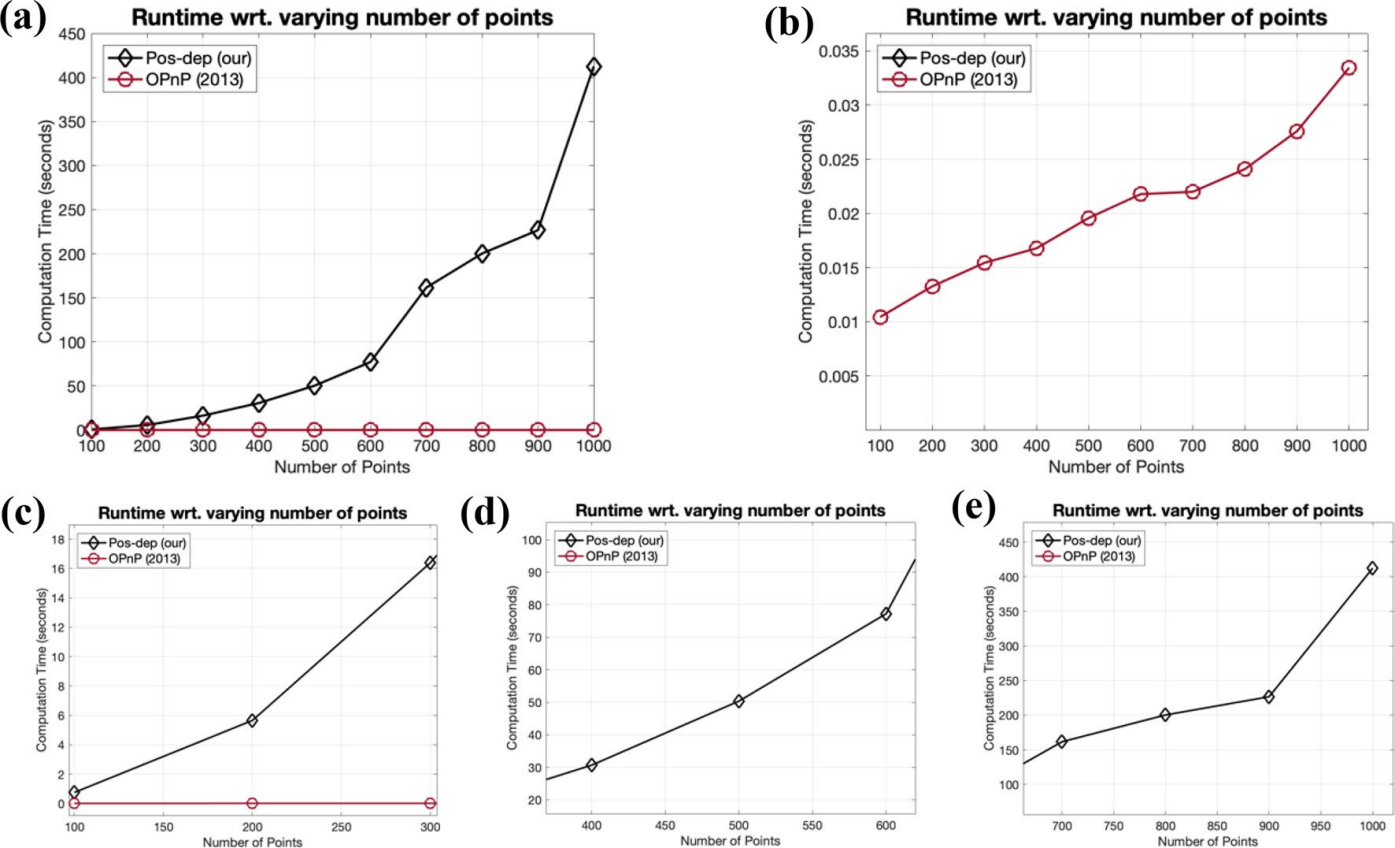
Runtime comparison between Pos-dep and OPnP when larger numbers of points are used. (**a**) Runtime comparison when the number of points ranges from 100 to 1000; (**b**) A close-up of the OPnP algorithm; (**c**) A close-up of the Pos-dep when the number of points equaling 100, 200, and 300; (**d**) A close-up of the Pos-dep when the number of points equaling 400, 500, and 600; (**e**) A close-up of the Pos-dep when the number of points equaling 700, 800, 900, and 1000.

In real applications, hundreds of or even thousands of points may exist. Therefore, we also studied the variations of computation time when the number of points is ranging from 100 to 1000, with a 100 increment. The runtime was recorded using MATLAB on the same PC with 16 GB RAM and 6-core Intel CPU with 2.6 GHz clock frequency. We compared our proposed algorithm with the more performant OPnP algorithm in Fig. 21.

We observe from Fig. 21 that similar to the case shown in Fig. 20, the runtime of Pos-dep increases with the increasing number of points. As discussed before, an increase of point numbers $n$ would cause an increase on the dimension of the matrix ${{\varvec{D}}}_{{\varvec{R}}}$, which is of size $2n\times 2n$. Thus, the eigenvalue and eigenvector computation of ${{\varvec{D}}}_{{\varvec{R}}}$ would be more computationally expensive. We also see the OPnP presents a slight runtime increase, ranging from approximately 0.01 s to over 0.03 s. We speculate such increase (only about 20 ms difference) was within the margin of system error caused by the heating-up machine with thermal throttling happening under prolonged computation process. Again, in Fig. 21 the averaged runtime over 100 independent runs at each point number was reported.

**Figure 22:**
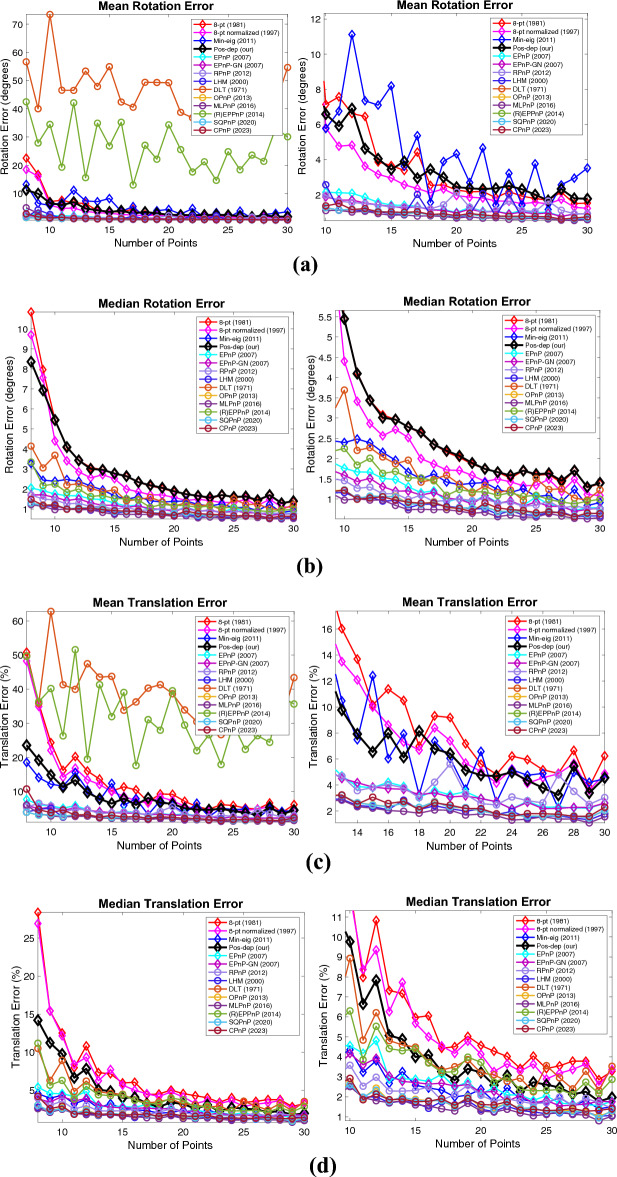

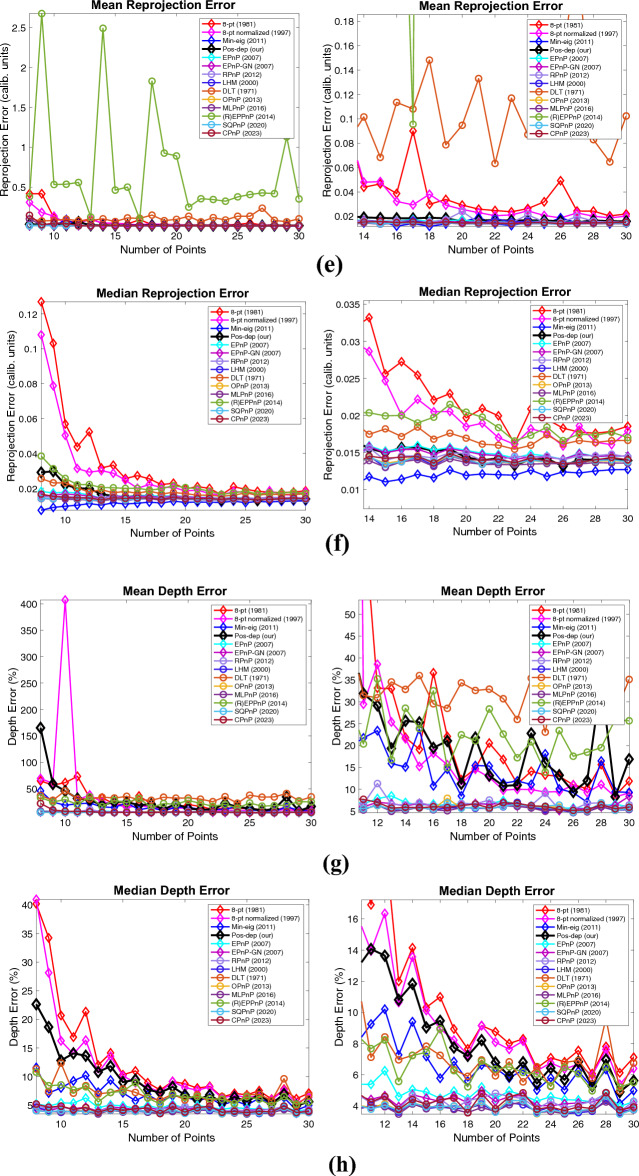
Mean and median rotation, translation, reprojection, and depth errors when varying the number of points under fixed noise of 8 pixels. (**a**) Mean rotation error, a close-up is shown to the right; (**b**) Median rotation error, a close-up is shown to the right; (**c**) Mean translation error, a close-up is shown to the right; (**d**) Median translation error, a close-up is shown to the right; (**e**) Mean reprojection error, a close-up is shown to the right; (**f**) Median reprojection error, a close-up is shown to the right; (**g**) Mean depth error, a close-up is shown to the right; (**h**) Median depth error, a close-up is shown to the right.

We noticed the thresholds chosen may be too small (such as 1e-14) for certain scenarios in the above experiments. Therefore, just like the first two scenarios, we also studied the mean and median estimation errors, which is thoroughly used in the OPnP work^20^. Also, in the above experiments, we did not add noise to the data when varying the number of points. While in the OPnP work^20^, zero-mean Gaussian noise with standard deviation of 2 pixels was added. Hence, we also added zero-mean Gaussian noise with a fixed standard deviation of 0.01 in calibrated image coordinates when studying the mean and median errors, which corresponds to noise with a standard deviation of 8 pixels. For each data point, 100 independent runs were performed, and the mean/median errors were reported. In^20^, 4 to 15 points with an increment of 1 are used; whereas in our experiments, to meet the requirement of each algorithm and to be consistent with the previous results, 8 to 30 points with an increment of 1 were used. The experimental results are shown in Fig. 22. The 5-pt and DLS were excluded from the graphs since they were out of bounds comparing to other algorithms.

For rotation errors, in Fig. 22a, when the point number reaches 13, the Pos-dep starts to yield a mean rotation error less than 5 degrees, which is considered the threshold of a good estimate in this study. In Fig. 22b, the median rotation error starts to fall below 5 degrees when the point number reaches 11. For translations, in Fig. 22c, when the point number reaches 13, the Pos-dep starts to yield a mean translation error less than 10%, which is considered the threshold of a good estimate in this study. In Fig. 22d, the median translation error starts to fall below 10% when the point number reaches 10. For reprojection errors, it can be seen from Fig. 22e and f that the Pos-dep was among the best, and when the point number reaches 14 and beyond, the Pos-dep reported an averaged reprojection error of less than 0.02 at each number of points. For depth errors, we see from Fig. 22g that the Pos-dep was better than (R)EPPnP; from Fig. 22h we see that when point number reaches 15, the Pos starts to yield median depth errors less 10%, which is considered the threshold of “good” estimate in this study. Through Fig. 22, we see the recent algorithms, the SQPnP and CPnP, both performed well, staying low among the algorithms across all point numbers.

### On real-world scenarios

The experiments on real-world data contain four scenarios/cases. The first and the second utilize standard datasets available online with ground truth provided^35^. The third case was conducted on a rigid box image taken from a mirrorless camera (SONY α6000). The fourth case was conducted on a satellite mockup image taken from an iPhone rear camera (iPhone SE2). Both cameras were self-calibrated using MATLAB’s camera calibration toolbox and a checkerboard pattern to obtain the intrinsic parameters.

## Results on the standard datasets

For the first scenario, we used ten pairs of successive dinosaur images from the dataset. We ran the testing for each image pair in the dataset and calculated the mean and median estimation errors. We removed the points not on the object and removed duplicate points, making the total number of correspondence points to be consistent (around ten) for each pair of images (we increased the number of points in the RANSAC experiment). These correspondences were generated by SIFT^36^ algorithm. We summarized the results in tabular forms and marked our algorithm in green. The images used from the Dino dataset can be found in Fig. S1 of the Supplementary Information.

We see from Table 1 that our algorithm reported an average of 0.5917 degrees, beating all algorithms except DLS, RPnP and OPnP; the median rotation error (0.6038) was below the 5-degree threshold for good estimates, being slightly better than the CPnP. The CPnP performed better than SQPnP.

**Table 1:** Rotation errors in degrees (Dino).

Image pairs	1&2	2&3	3&4	4&5	5&6	6&7	7&8	8&9	9&10	10&11	Mean	Median
8-pt	7.8884	8.0363	7.6181	7.7893	7.9252	7.8242	7.9051	9.9923	7.8812	5.7277	7.8588	7.8848
8-pt (norm)	8.2328	6.4916	7.2263	7.8695	7.7816	7.7607	7.6832	5.9446	7.8766	5.0236	7.1891	7.7219
Min-eig	3.6851	7.4006	7.4038	3.5986	18.2585	8.1787	9.1135	10.2902	35.8349	18.1595	12.1923	8.6461
Pos-dep	0.4347	0.5406	0.6898	0.2762	0.3244	0.2202	0.7644	0.6670	0.9341	1.0659	**0.5917**	**0.6038**
5-pt	7.6648	7.6234	75.4526	7.7430	7.6878	7.3212	10.1215	6.5036	68.1847	6.4833	20.4786	7.6763
EPnP	1.3631	2.0597	0.7881	0.7417	7.3072	3.5389	1.9125	1.8633	0.9640	5.4932	2.6032	1.8879
EPnP-GN	1.9144	7.6234	0.7881	0.1015	0.5285	0.3899	1.9125	0.0960	0.9640	2.3202	0.9333	0.8760
DLS	0.1358	0.2317	0.0735	0.0777	0.2587	0.0573	0.4345	0.0361	0.0971	0.2984	0.1701	0.1164
RPnP	0.0782	0.2646	0.5915	0.1532	0.3032	0.0599	0.4107	0.1118	0.1021	0.2268	0.2302	0.1900
LHM	13.8819	15.5750	21.4551	22.0445	18.7450	19.6999	17.0439	14.6192	9.0274	0.6153	15.2707	16.3094
DLT	179.2787	179.4312	179.8910	0.2405	34.5961	179.5832	1.9966	179.7438	179.4126	6.8974	112.1071	179.3456
OPnP	0.1357	0.2315	0.0734	0.0772	0.2590	0.0568	0.4330	0.0363	0.0962	0.3012	0.1700	0.1159
MLPnP	0.1345	0.2260	0.0745	0.0765	103.4802	0.0558	0.4299	0.0369	103.4803	61.4937	26.9488	0.1803
(R)EPPnP	0.3472	1.6942	1.1462	1.2608	178.5284	0.6232	7.6186	178.2010	36.9589	17.8710	42.4250	4.6564
SQPnP	1.6267	1.6253	1.1790	3.4899	11.2844	2.9392	3.2843	1.6422	7.3630	30.0866	6.4521	3.1117
CPnP	0.1482	0.3478	0.0708	1.0026	22.8059	0.2081	0.9658	0.0455	1.8607	9.0728	3.6528	0.6568

Significant values are in bold.

For translations, from Table 2 we see our Pos-dep reported 5.2030% for translation error, outperforming all except the DLS, RPnP and OPnP. The median error (4.4400%) was below the 10% threshold for good estimates. This time, the SQPnP performed better than the CPnP in both mean and median errors.

**Table 2:** Translation errors in percentage (Dino).

Image pairs	1&2	2&3	3&4	4&5	5&6	6&7	7&8	8&9	9&10	10&11	Mean	Median
8-pt	147.9800	143.9900	137.7300	140.8600	148.9800	147.7700	139.9700	129.9900	137.3400	122.6800	139.7290	140.4150
8-pt (norm)	159.2400	129.6500	134.4900	142.7500	149.6700	147.8900	134.0200	6.3200	137.2900	118.5800	125.9900	135.8900
Min-eig	13.5500	139.0200	115.6400	2.0400	49.4500	196.7100	35.7400	199.8900	96.0800	47.2500	89.5370	72.7650
Pos-dep	4.2800	3.1100	4.6000	2.3000	4.6000	1.1900	3.9600	8.5000	6.0800	13.4100	**5.2030**	**4.4400**
5-pt	161.9300	133.6100	159.3300	137.7600	138.9000	115.4000	41.0900	19.0900	74.3700	69.0600	105.0540	124.5050
EPnP	12.1800	24.5600	9.9100	5.1200	60.8200	32.9800	25.2300	9.5400	11.6900	37.7500	22.9780	18.3700
EPnP-GN	3.5500	23.5400	9.9100	1.2800	7.2600	4.9600	25.2300	1.0700	11.6900	30.6600	11.9150	8.5850
DLS	1.7500	1.8300	0.4600	0.8500	2.0500	0.6400	4.0800	0.4200	0.7400	3.9800	1.6800	1.3000
RPnP	0.7800	3.8700	6.4800	2.0100	2.3100	0.7100	4.5800	1.5200	0.6600	3.0700	2.5990	2.1600
LHM	31.8600	84.6500	92.3000	105.6400	133.2500	131.4300	146.8700	100.3700	138.2300	7.7900	97.2390	103.0050
DLT	174.4600	166.0000	161.6700	2.4700	73.0300	138.8700	26.0700	129.6100	97.7900	40.9300	101.0900	113.7000
OPnP	1.7500	1.8200	0.4600	0.8500	2.0400	0.6300	4.0600	0.4200	0.7300	4.0100	1.6770	1.3000
MLPnP	1.7300	1.7400	0.4600	0.8400	132.3400	0.6000	4.0500	0.4400	143.9700	189.1200	47.5290	1.7350
(R)EPPnP	4.8300	20.9900	13.2000	20.6000	154.3900	12.6200	66.4400	165.9100	92.7400	84.6500	63.6370	43.7150
SQPnP	17.9700	21.4700	13.6600	46.2800	49.7100	28.7100	40.2900	5.8700	114.5800	183.5800	52.2120	34.5000
CPnP	2.8300	5.3900	1.5300	94.1500	148.5300	19.5200	69.3600	2.0200	92.6100	105.5700	54.1510	44.4400

Significant values are in bold.

We can see from Table 3 that the reprojection error of our Pos-dep was 0.0001, which is the smallest among all algorithms and matches the OPnP. The median error of Pos-dep (0.0001) was also small compared to other algorithms. The SQPnP had smaller reprojection errors in both mean and median than the CPnP.

**Table 3:** Reprojection errors in calibrated units (Dino).

Image pairs	1&2	2&3	3&4	4&5	5&6	6&7	7&8	8&9	9&10	10&11	Mean	Median
8-pt	0.0035	0.0071	0.0032	0.0302	0.0024	0.0024	0.0012	0.0551	0.0039	0.0116	0.0121	0.0037
8-pt (norm)	0.0066	0.0081	0.0062	0.0055	0.0075	0.0020	0.0253	0.0070	0.0038	0.0087	0.0081	0.0068
Min-eig	0	0.0001	0.0001	0	0	0	0	0	0.0002	0.0001	0.0001	0
Pos-dep	0	0.0001	0.0003	0.0001	0	0.0001	0.0001	0	0.0001	0.0004	**0.0001**	**0.0001**
5-pt	0.0001	0.0005	0.0009	0.0010	0.0003	0.0001	0	0	0.0004	0.0003	0.0004	0.0003
EPnP	0.0002	0.0002	0.0001	0.0001	0.0004	0.0002	0.0001	0.0001	0.0002	0.0016	0.0003	0.0002
EPnP-GN	0	0.0003	0.0001	0	0	0.0001	0.0001	0	0.0002	0.0007	0.0002	0.0001
DLS	0	0.0001	0	0	0	0	0.0001	0	0.0001	0.0003	0.0001	0
RPnP	0	0.0001	0.0001	0	0	0	0.0001	0	0.0001	0.0003	0.0001	0.0001
LHM	0.0008	0.0012	0.0014	0.0012	0.0007	0.0011	0.0010	0.0007	0.0019	0.0003	0.0010	0.0011
DLT	0.0003	0.0004	0.0003	0	0.0018	0.0005	0.0001	0.0007	0.0021	0.0015	0.0008	0.0005
OPnP	0	0.0001	0	0	0	0	0.0001	0	0.0001	0.0003	0.0001	0
MLPnP	0	0.0001	0	0	0.1868	0	0.0001	0	2.5988	0.2616	0.3047	0.0001
(R)EPPnP	0.0002	0.0008	0.0001	0.0040	0.0182	0.0042	0.0013	0.0460	0.0142	0.0062	0.0095	0.0041
SQPnP	0.0004	0.0002	0.0001	0.0003	0.0007	0.0001	0.0003	0.0001	0.0016	0.0022	0.0006	0.0003
CPnP	0.0007	0.0012	0.0001	0.0485	0.4328	0.0042	0.0342	0.0004	0.0528	0.2378	0.0813	0.0192

Significant values are in bold.

For depth errors, we rounded the results to integers. We can see from Table 4 that both the mean and median errors of our Pos-dep were 4%, only larger than the DLS, RPnP and OPnP algorithms, and were comparable to the EPnP-GN. The SQPnP had smaller mean depth error than the CPnP, while its median error was larger than CPnP.

**Table 4:** Depth errors in percentage (Dino).

Image pairs	1&2	2&3	3&4	4&5	5&6	6&7	7&8	8&9	9&10	10&11	Mean	Median
8-pt	66	133	62	168	714	386	209	80	36	40	189	107
8-pt (norm)	128	16	35	222	213	222	116	330	37	42	136	122
Min-eig	91	340	1625	90	69	1365	52	181	80	69	396	91
Pos-dep	3	7	1	2	0	1	9	4	10	4	**4**	**4**
5-pt	5217	1256	89	4016	383	979	54	609	89	395	1309	502
EPnP	16	16	2	8	37	55	6	28	7	38	21	16
EPnP-GN	3	8	2	0	1	2	6	1	7	11	4	3
DLS	0	2	1	0	3	0	3	0	1	0	1	1
RPnP	1	1	4	0	3	0	2	0	1	0	1	1
LHM	64	61	69	69	58	61	50	56	31	2	52	60
DLT	100	100	100	2	80	100	7	100	100	44	73	100
OPnP	0	2	1	0	3	0	3	0	1	0	1	1
MLPnP	0	2	1	0	93	0	3	0	107	108	31	2
(R)EPPnP	4	6	6	3	100	4	41	101	83	69	42	24
SQPnP	9	1	6	5	58	46	13	27	40	68	27	20
CPnP	1	2	1	46	109	1	26	1	33	132	35	14

Significant values are in bold.

In terms of negative depth instances, by checking the experimental data we see that out of ten pairs of images, negative depths occurred in two image pairs for the (R)EPPnP, three image pairs for the 8-pt (norm), four image pairs for the 5-pt, six image pairs for the DLT and the 8-pt algorithms. All other algorithms, including our Pos-dep, recorded zero negative instances.

For the Dino dataset, the computation time of each algorithm on each image pair, is summarized in Table 5. We see the Pos-dep yielded 0.0066 s (6.6 ms) for both the mean and median computation time on single image pair, which is comparable to the SQPnP algorithm, which yielded 6.5 ms for the mean time, and 6.8 ms for the median time. The computation time of SQPnP was nearly half of that of CPnP.

**Table 5:** Computation time on each image pair (Dino).

Image pairs	1&2	2&3	3&4	4&5	5&6	6&7	7&8	8&9	9&10	10&11	Mean	Median
8-pt	0.0128	0.0170	0.0128	0.0132	0.0182	0.0122	0.0130	0.0130	0.0129	0.0131	0.0138	0.0130
8-pt (norm)	0.0353	0.0419	0.0344	0.0341	0.0371	0.0332	0.0341	0.0345	0.0342	0.0362	0.0355	0.0345
Min-eig	0.1465	0.1352	0.0963	0.1197	0.0974	0.0939	0.0950	0.0899	0.1122	0.1097	0.1096	0.1036
Pos-dep	0.0057	0.0082	0.0052	0.0067	0.0083	0.0078	0.0064	0.0077	0.0053	0.0044	**0.0066**	**0.0066**
5-pt	0.0607	0.0715	0.0517	0.0570	0.0662	0.0615	0.0640	0.0509	0.0480	0.0481	0.0580	0.0588
EPnP	0.0216	0.0302	0.0215	0.0214	0.0278	0.0220	0.0212	0.0214	0.0218	0.0210	0.0230	0.0215
EPnP-GN	0.0342	0.0417	0.0334	0.0331	0.0407	0.0346	0.0329	0.0343	0.0353	0.0346	0.0355	0.0344
DLS	0.0558	0.0594	0.0577	0.0571	0.0617	0.0552	0.0543	0.0529	0.0560	0.0517	0.0562	0.0559
RPnP	0.0316	0.0347	0.0311	0.0383	0.0356	0.0338	0.0325	0.0321	0.0345	0.0342	0.0338	0.0340
LHM	0.0282	0.0259	0.0257	0.0267	0.0291	0.0278	0.0254	0.0228	0.0253	0.0212	0.0258	0.0258
DLT	0.0107	0.0084	0.0078	0.0119	0.0083	0.0073	0.0076	0.0085	0.0085	0.0068	0.0086	0.0083
OPnP	0.0897	0.0738	0.0676	0.0874	0.0743	0.0542	0.0775	0.0518	0.0832	0.0698	0.0729	0.0741
MLPnP	0.1000	0.1006	0.0980	0.1053	0.0989	0.1032	0.1032	0.1021	0.0975	0.0853	0.0994	0.1003
(R)EPPnP	0.0172	0.0209	0.0202	0.0166	0.0197	0.0221	0.0173	0.0190	0.0183	0.0161	0.0187	0.0186
SQPnP	0.0064	0.0073	0.0070	0.0063	0.0058	0.0069	0.0068	0.0069	0.0068	0.0052	0.0065	0.0068
CPnP	0.0130	0.0134	0.0147	0.0123	0.0144	0.0132	0.0135	0.0129	0.0142	0.0116	0.0133	0.0133

Significant values are in bold.

For the second scenario, we tested the Temple dataset from the same website^35^. Please also refer to pages 227–234 of this thesis^34^. Recent algorithms such as SQPnP and CPnP were again included. Again, successive image pairs were used. We ran the testing for each image pair in the dataset and calculated the mean and median estimation errors. We removed the points not on the object and removed duplicate points, making the total number of correspondence points to be consistent (around ten) for each pair of images (we increased the number of points in the RANSAC experiment). These correspondences were generated by SIFT^36^ algorithm. The results are summarized in tabular forms. The images used from the Temple dataset can be found in Fig. S2 of the Supplementary Information.

We see from Table 6 that our algorithm reported an average of 1.3111 degrees, beating all algorithms except DLS, EPnP-GN and OPnP; the median rotation error (1.0991) was below the 5-degree threshold for good estimates, and was better than SQPnP. CPnP performed better than SQPnP in both mean and median rotation errors.

**Table 6:** Rotation errors in degrees (Temple).

Image pairs	1&2	2&3	3&4	4&5	5&6	6&7	7&8	8&9	9&10	10&11	Mean	Median
8-pt	4.8508	2.0432	7.3010	7.5987	12.4694	6.3913	7.7135	8.1877	9.8206	6.4653	7.2842	7.4498
8-pt (norm)	4.5275	1.3265	0.9927	1.3891	12.4049	5.3872	4.0223	8.1941	5.0213	6.2467	4.9512	4.7744
Min-eig	0.9115	9.9877	2.4766	9.1484	0.3100	8.2440	12.9311	60.1822	2.4051	19.6816	12.6278	8.6962
Pos-dep	2.4509	0.9282	1.0306	0.8991	0.7952	1.1677	1.3730	1.5160	2.4136	0.5372	**1.3111**	**1.0991**
5-pt	1.2780	51.6307	8.7866	8.0247	3.3846	8.2250	8.1138	3.6024	0.8918	7.6494	10.1587	7.8370
EPnP	0.5461	1.1246	1.2564	0.1807	3.2481	1.5711	1.4594	5.6405	3.7114	4.0392	2.2777	1.5152
EPnP-GN	0.0247	0.0742	0.1021	0.1807	0.4659	0.3485	1.4594	1.9252	0.8035	1.0302	0.6414	0.4072
DLS	0.0268	0.0557	0.3243	0.2526	0.2580	0.4445	1.3559	1.1826	1.0255	0.4037	0.5330	0.3640
RPnP	0.0448	0.0832	1.4202	0.1356	0.5048	0.3986	1.9210	1.2298	2.1148	0.4430	0.8296	0.4739
LHM	1.5326	0.7413	1.2727	0.6868	3.8927	5.6404	1.9088	2.4828	2.6273	3.7991	2.4585	2.1958
DLT	0.5465	179.8900	179.7759	179.9453	179.9914	179.8598	179.5160	179.7782	3.7307	6.2678	126.9302	179.7771
OPnP	0.0261	0.0556	0.3274	0.2533	0.2576	0.4437	1.3546	1.1828	1.0234	0.3934	0.5318	0.3604
MLPnP	0.0214	0.0477	0.3446	0.2550	0.2544	0.4348	1.3239	139.8008	1.0064	0.4056	14.3895	0.3751
(R)EPPnP	179.9103	179.8130	176.9715	1.7407	179.7147	178.8965	178.8356	178.1579	176.1635	177.7765	160.7980	178.4968
SQPnP	0.0260	0.0555	0.9662	0.9551	2.4492	2.0008	1.7471	9.1880	4.5981	0.4041	2.2390	1.3567
CPnP	0.0196	0.0555	0.3349	0.2689	0.2251	0.4361	1.3881	10.4874	0.9182	7.2368	2.1371	0.3855

Significant values are in bold.

For translations, from Table 7 we see our Pos-dep reported 8.9336% for translation error, outperforming all except the DLS, EPNP-GN and OPnP. The median error (9.9931%) was below the 10% threshold. The SQPnP performed better than the CPnP in mean error, while worse than CPnP in median error.

**Table 7:** Translation errors in percentage (Temple).

Image pairs	1&2	2&3	3&4	4&5	5&6	6&7	7&8	8&9	9&10	10&11	Mean	Median
8-pt	92.1422	79.9670	138.5780	143.4303	168.6506	51.6684	146.1642	143.2984	88.6514	122.0048	117.4545	130.2914
8-pt (norm)	92.3444	68.8238	33.0956	11.8773	168.3752	40.8675	35.3483	143.2532	89.3272	120.9617	80.4274	79.0755
Min-eig	1.3116	199.9352	3.1606	199.4768	3.3325	194.8457	47.1561	77.1796	17.6167	67.2470	81.1262	57.2015
Pos-dep	1.2713	0.4665	11.1998	5.2681	8.7865	12.3866	16.0186	16.4625	12.2092	5.2673	**8.9336**	**9.9931**
5-pt	4.5532	194.9352	48.5920	97.7144	39.8143	109.4196	156.9949	16.4985	1.9206	71.1663	74.1609	59.8792
EPnP	1.9476	5.9481	13.9360	2.1803	24.2348	3.6684	18.0885	63.8242	40.6849	48.5964	22.3109	16.0123
EPnP-GN	0.3517	1.2242	1.7413	2.1803	5.8258	4.3889	18.0885	23.0646	10.1091	13.1372	8.0112	5.1074
DLS	0.3640	0.6861	3.8504	2.8801	3.2150	5.5473	16.9692	14.9109	12.6214	5.1926	6.6237	4.5215
RPnP	0.5936	1.1740	17.8651	0.2945	6.1954	4.9625	23.9736	15.9794	25.3246	5.5783	10.1941	5.8868
LHM	20.1749	9.7372	16.1284	7.7855	41.7067	61.3198	22.9370	30.6949	31.2081	44.8566	28.6549	26.8160
DLT	1.9519	90.0492	162.5359	172.1880	149.9793	37.5055	123.5760	114.6655	40.8082	25.6747	91.8934	102.3573
OPnP	0.3567	0.6887	3.8923	2.8853	3.2102	5.5367	16.9519	14.9248	12.5996	5.0539	6.6100	4.4731
MLPnP	0.3082	0.5765	4.1369	2.9042	3.1703	5.4240	16.5627	39.6514	12.3860	5.2180	9.0338	4.6775
(R)EPPnP	137.8186	107.8105	92.2194	20.2228	111.6444	86.2589	87.6170	80.5449	144.1819	152.8809	102.1199	100.0149
SQPnP	0.3600	0.6800	11.2200	1.6700	17.9200	2.6800	13.5100	156.2700	22.3000	5.2000	23.1810	8.2100
CPnP	0.3700	2.9500	7.7500	5.2200	2.8800	5.7200	21.4500	142.1600	34.7400	121.7500	34.4990	6.7350

Significant values are in bold.

We can see from Table 8 that the reprojection error of our Pos-dep was 0.0005, which was among the smallest ones. It was comparable to SQPnP and better than CPnP. The median error of Pos-dep (0.0003) was also among the best, and better than the CPnP. The SQPnP had smaller reprojection errors in both mean and median errors than the CPnP.

**Table 8:** Reprojection errors in calibrated units (Temple).

Image pairs	1&2	2&3	3&4	4&5	5&6	6&7	7&8	8&9	9&10	10&11	Mean	Median
8-pt	0.0087	0.0098	0.0184	0.0397	0.0090	0.0225	0.0130	0.0008	0.0577	0.0140	0.0194	0.0135
8-pt (norm)	0.0165	0.0069	0.0064	0.0008	0.0247	0.0028	0.0229	0.0008	0.0278	0.0136	0.0123	0.0102
Min-eig	0	0.0004	0.0001	0.0003	0	0.0001	0.0003	0.0008	0.0002	0.0006	0.0003	0.0003
Pos-dep	0.0017	0.0002	0.0002	0.0002	0.0001	0.0002	0.0003	0.0008	0.0004	0.0004	**0.0005**	**0.0003**
5-pt	0.0001	0.0021	0.0006	0.0003	0.0002	0.0004	0.0004	0.0005	0.0002	0.0006	0.0005	0.0004
EPnP	0.0002	0.0008	0.0003	0.0001	0.0001	0.0001	0.0003	0.0011	0.0004	0.0009	0.0004	0.0003
EPnP-GN	0.0001	0.0001	0.0003	0.0001	0.0001	0.0001	0.0003	0.0008	0.0005	0.0009	0.0003	0.0002
DLS	0.0001	0.0001	0.0001	0.0001	0	0.0001	0.0002	0.0006	0.0003	0.0004	0.0002	0.0001
RPnP	0.0001	0.0001	0.0002	0.0001	0	0.0001	0.0002	0.0009	0.0003	0.0004	0.0002	0.0002
LHM	0.0008	0.0004	0.0003	0.0001	0.0003	0.0009	0.0004	0.0008	0.0004	0.0010	0.0005	0.0004
DLT	0.0002	0.0125	0.0032	0.0009	0.0010	0.0037	0.0027	0.0042	0.0004	0.0009	0.0030	0.0019
OPnP	0.0001	0.0001	0.0001	0.0001	0	0.0001	0.0002	0.0006	0.0003	0.0004	0.0002	0.0001
MLPnP	0.0001	0.0001	0.0001	0.0001	0	0.0001	0.0002	0.2539	0.0003	0.0005	0.0255	0.0001
(R)EPPnP	0.0608	0.0036	0.1171	0.0005	0.1303	0.0538	0.0870	0.0489	0.0943	0.0398	0.0636	0.0573
SQPnP	0.0001	0.0001	0.0002	0.0001	0.0001	0.0001	0.0002	0.0023	0.0004	0.0004	0.0004	0.0002
CPnP	0.0001	0.0003	0.0009	0.0005	0.0001	0.0001	0.0017	0.0452	0.0048	0.1265	0.0180	0.0007

Significant values are in bold.

For depth errors, we rounded the results to integers. We can see from Table 9 that both the mean and median errors of our Pos-dep were 4%. The mean error of Pos-dep beat those of both the SQPnP and CPnP. The median error of Pos-dep beat that of SQPnP. The CPnP performed better than the SQPnP in terms of both mean and median depth errors.

**Table 9:** Depth errors in percentage (Temple).

Image pairs	1&2	2&3	3&4	4&5	5&6	6&7	7&8	8&9	9&10	10&11	Mean	Median
8-pt	20	12	30	35	13	170	86	1267	62	15	171	33
8-pt (norm)	14	11	4	11	9	183	9	1020	59	16	134	13
Min-eig	13	292	45	614	1	21,021	59	87	33	68	2223	64
Pos-dep	4	10	1	1	5	3	6	7	4	2	**4**	**4**
5-pt	13	82	47	20,276	7	7674	3139	73	12	1937	3326	78
EPnP	7	13	10	1	55	24	5	14	13	88	15	13
EPnP-GN	0	1	1	1	1	0	5	4	2	3	2	1
DLS	0	0	1	1	0	0	1	1	1	0	1	1
RPnP	0	0	1	2	1	0	0	2	4	1	1	1
LHM	3	1	2	3	15	18	5	2	6	9	6	4
DLT	7	101	100	100	100	117	100	101	13	41	78	100
OPnP	0	0	1	1	0	0	1	1	1	0	1	1
MLPnP	0	0	1	1	0	0	1	88	1	1	9	1
(R)EPPnP	102	100	100	6	101	100	100	98	102	99	91	100
SQPnP	0	0	1	11	36	33	22	159	34	0	30	17
CPnP	0	1	1	1	0	0	1	132	3	55	19	1

Significant values are in bold.

In terms of negative depth instances, by checking the experimental data we see that out of ten pairs of images, negative depths occurred in one image pair for the 8-pt (norm) and CPnP, three image pairs for the 8-pt, four image pairs for the 5-pt, eight image pairs for the DLT, nine image pairs for the (R)EPPnP. All other algorithms, including our Pos-dep, recorded zero negative instances.

For the Temple dataset, the computation time of each algorithm on each image pair, is summarized in Table 10. We see the Pos-dep yielded 0.0040 s (4 ms) for the mean time, and 0.0036 s (3.6 ms) for the median time. The Pos-dep took less computation time than both the more recent SQPnP and CPnP algorithms. Again, we found the computation time of SQPnP was nearly half of that of CPnP.

**Table 10:** Computation time on each image pair (Temple).

Image pairs	1&2	2&3	3&4	4&5	5&6	6&7	7&8	8&9	9&10	10&11	Mean	Median
8-pt	0.0143	0.0148	0.0134	0.0146	0.0140	0.0166	0.0178	0.0139	0.0141	0.0144	0.0148	0.0143
8-pt (norm)	0.0573	0.0361	0.0342	0.0358	0.0450	0.0413	0.0447	0.0371	0.0339	0.0361	0.0402	0.0366
Min-eig	0.1582	0.0898	0.1176	0.0885	0.1240	0.1017	0.0952	0.0972	0.0906	0.0936	0.1056	0.0962
Pos-dep	0.0017	0.0030	0.0021	0.0063	0.0027	0.0029	0.0074	0.0057	0.0043	0.0041	**0.0040**	**0.0036**
5-pt	0.0491	0.0625	0.0526	0.0509	0.0467	0.0432	0.0438	0.0510	0.0668	0.0452	0.0512	0.0500
EPnP	0.0205	0.0257	0.0201	0.0197	0.0199	0.0200	0.0233	0.0198	0.0236	0.0269	0.0220	0.0203
EPnP-GN	0.0339	0.0328	0.0323	0.0321	0.0326	0.0339	0.0341	0.0316	0.0428	0.0429	0.0349	0.0334
DLS	0.0500	0.0502	0.0504	0.0508	0.0539	0.0503	0.0506	0.0510	0.0623	0.0550	0.0525	0.0507
RPnP	0.0272	0.0295	0.0242	0.0236	0.0314	0.0279	0.0310	0.0270	0.0300	0.0241	0.0276	0.0275
LHM	0.0216	0.0249	0.0201	0.0213	0.0240	0.0209	0.0236	0.0211	0.0233	0.0225	0.0223	0.0221
DLT	0.0072	0.0075	0.0069	0.0067	0.0087	0.0071	0.0071	0.0071	0.0090	0.0073	0.0075	0.0072
OPnP	0.0524	0.0483	0.0538	0.0499	0.0628	0.0466	0.0658	0.0467	0.0723	0.0464	0.0545	0.0512
MLPnP	0.0763	0.0818	0.0727	0.0729	0.0773	0.0745	0.0748	0.0783	0.0820	0.0778	0.0768	0.0768
(R)EPPnP	0.0166	0.0186	0.0164	0.0160	0.0180	0.0166	0.0176	0.0131	0.0173	0.0156	0.0166	0.0166
SQPnP	0.0054	0.0064	0.0052	0.0057	0.0063	0.0055	0.0074	0.0053	0.0059	0.0052	0.0058	0.0056
CPnP	0.0126	0.0155	0.0125	0.0121	0.0136	0.0121	0.0121	0.0122	0.0136	0.0123	0.0129	0.0124

Significant values are in bold.

### Results on the rigid box image

For the third scenario, we performed the experiment on a rigid box. To get the 3D-2D matches, we used a SONY α6000 mirrorless camera with a fixed focal length of 30 mm to complete the calibration process using a checkerboard pattern. Then, we used the same calibrated camera without refocusing to capture the image of the rigid box. The original dimension of the image was 6000 (H) by 4000 (V) pixels. We chose ten correspondence points on the surface of the rigid box with MATLAB’s “getpts” control point selection functionality for 2D point selection. Since the ground truth was not known, we transformed the two 3D-2D problem into one 3D-3D problem with ground truth known. The rigid box image with control points (correspondences) selected, as well as the transformation method from 3D-2D to 3D-3D, are presented in Figs. S3 and S4 of Supplementary Information.

**Figure 23:**
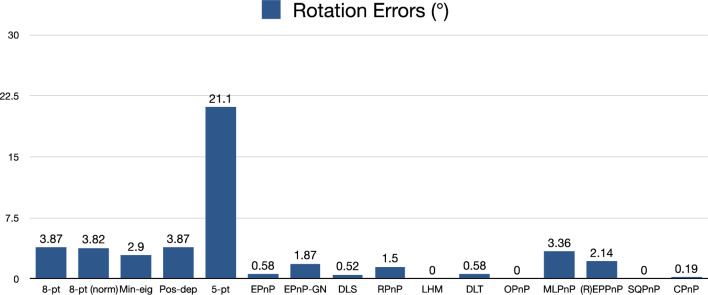
Rotation errors in degrees (rigid box).

For rotation errors, we see from Fig. 23 that our Pos-dep was 3.87 degrees, comparable to the 8pt, normalized 8-pt, and MLPnP. The SQPnP and OPnP were at zero errors, better than the CPnP (0.19 degrees).

**Figure 24:**
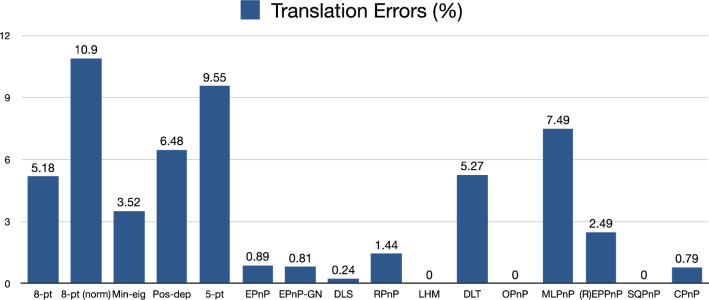
Translation errors in percentage (rigid box).

We see from Fig. 24 that the Pos-dep had good translation errors of 6.48%, lower than the 10% threshold and better than the normalized 8-pt, 5-pt, and MLPnP; the LHM, OPnP, and SQPnP had zero errors, better than the CPnP (0.79%).

**Figure 25:**
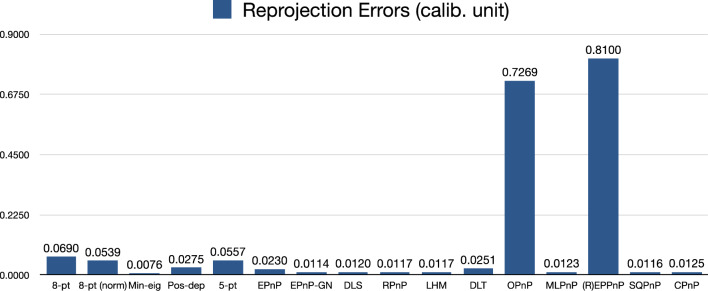
Reprojection errors in calibrated units (rigid box).

We can see from Fig. 25 that the reprojection error of our Pos-dep was 0.0275, among the smallest and comparable to EPnP’s. The SQPnP and CPnP had similar reprojection errors (0.0116, 0.0125) with the SQPnP performed slightly better.

**Figure 26:**
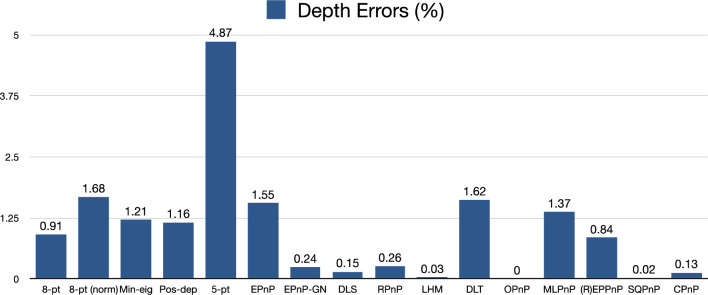
Depth errors in percentage (rigid box).

From Fig. 26, we see the depth error of Pos-dep was 1.16%, much lower than the 10% threshold for good estimates. The SQPnP (0.02%) performed better than the CPnP (0.13%) algorithm in terms of depth errors.

In the rigid box case, no negative depths were reported for all algorithms. In terms of computation time, the 8-pt was 0.0078 s, the 8-pt norm 0.0282 s, the Min-eig 0.0417 s, the Pos-dep 0.0100 s, the 5-pt 0.0147 s, the EPnP 0.1177 s, the EPnP-GN 0.0155 s, the DLS 0.0322 s, the RPnP 0.0050 s, the LHM 0.0047 s, the DLT 0.0016 s, the OPnP 0.0340 s, the MLPnP 0.0131 s, the (R)EPPnP 0.0083 s, the SQPnP 0.0026 s, and the CPnP was 0.0034 s. The 10 ms-computation time of Pos-dep was most close to those of the MLPnP (13.1 ms) and (R)EPPnP (8.3 ms). The SQPnP (2.6 ms) had less computation time than the CPnP (3.4 ms).

Using the estimated results, it is tempting to calculate the 3D reconstructed model of the rigid box and compare it with the original one. This result is included in Fig. S5 of Supplementary Information.

### Results on the satellite mockup image

For the fourth scenario, we performed the experiment on a satellite mockup image. The process to obtain the 3D-2D matches was similar to that in the previous rigid box case. The original dimension of the image this time was 4032 (H) by 3024 (V) pixels. The focal length was fixed at 3.99 mm on the iPhone’s rear-facing camera throughout the calibration process. Since the ground truth was again not known, we applied the same 3D-2D to 3D-3D transform as that used in the rigid box case. The configuration of the satellite mockup with coordinate system setup and selected correspondence points, was shown in Fig. S6 of Supplementary Information.

**Figure 27:**
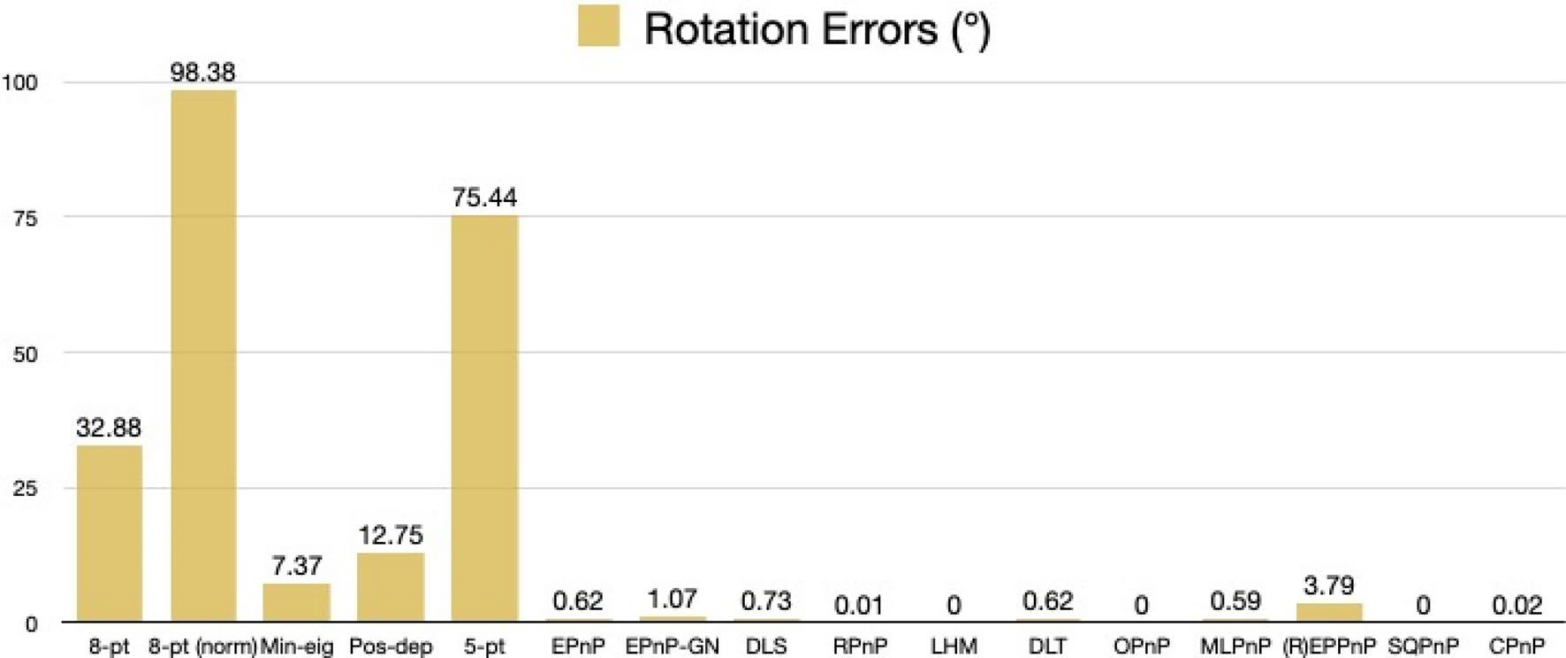
Rotation errors in degrees (satellite mockup).

For rotation errors, we see from Fig. 27 that our Pos-dep reported 12.75 degrees. The SQPnP, LHM, and OPnP were at zero errors, better than the CPnP (0.02 degrees).

**Figure 28:**
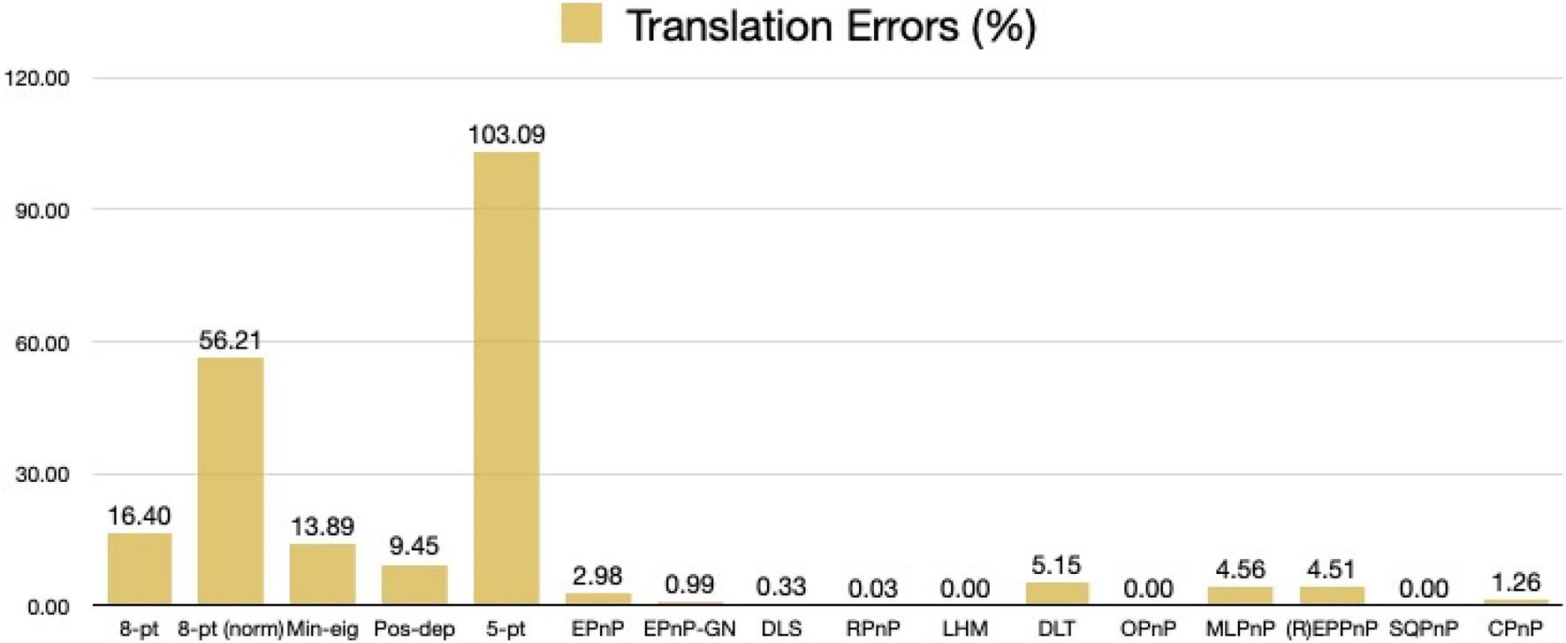
Translation errors in percentage (satellite mockup).

We see from Fig. 28 that the Pos-dep reported a translation error of 9.45%, lower than the 10% threshold; the LHM, OPnP, and SQPnP had zero errors, better than the CPnP (1.26%).

**Figure 29:**
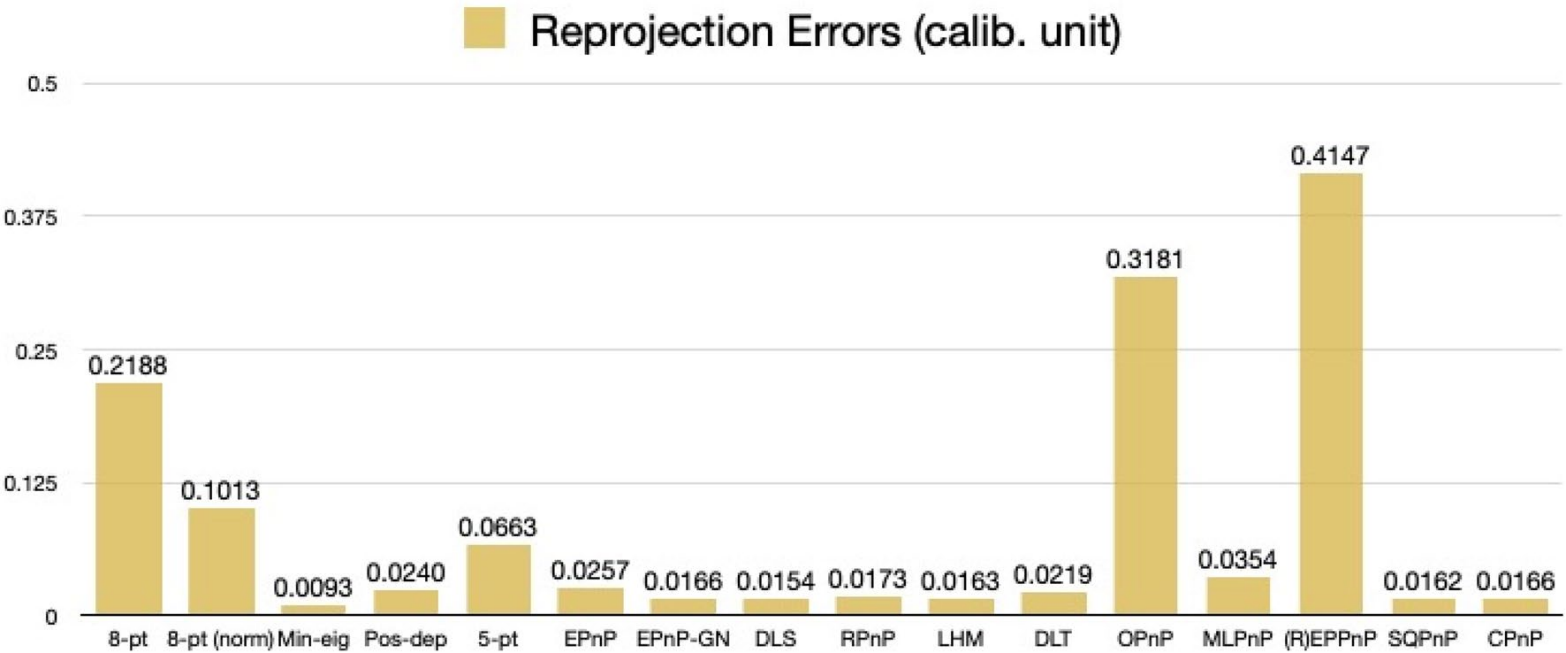
Reprojection errors in calibrated units (satellite mockup).

We can see from Fig. 29 that the reprojection error of our Pos-dep was 0.0240, among the smallest ones and comparable to EPnP. The SQPnP and CPnP had similar reprojection errors (0.0162, 0.0166) with the SQPnP performed slightly better.

**Figure 30:**
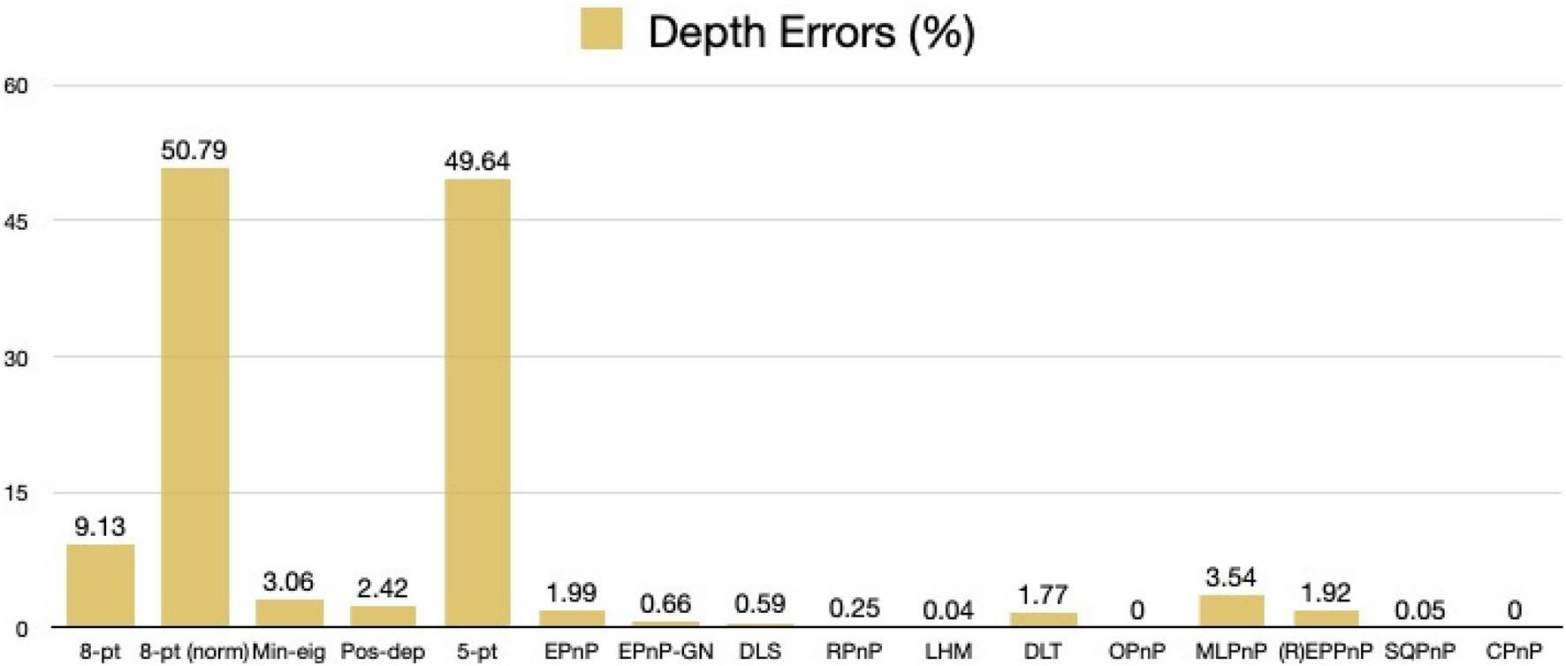
Depth errors in percentage (satellite mockup).

From Fig. 30, we see the depth error of Pos-dep was 2.42%, much lower than the 10% threshold for good estimates. The CPnP (0%) was better than the SQPnP (0.05%) in terms of depth errors.

In the satellite mockup case, all algorithms except the 5-pt reported positive depths. In terms of computation time, the 8-pt was 0.0006 s, the 8-pt norm 0.0078 s, the Min-eig 0.0498 s, the Pos-dep 0.0103 s, the 5-pt 0.0037 s, the EPnP 0.0103 s, the EPnP-GN 0.0102 s, the DLS 0.0029 s, the RPnP 0.0025 s, the LHM 0.0034 s, the DLT 0.0005 s, the OPnP 0.0199 s, the MLPnP 0.0055 s, the (R)EPPnP 0.0024 s, the SQPnP 0.0023 s, and the CPnP was 0.0010 s. The 10.3 ms-computation time of Pos-dep was most close to those of the EPnP (10.3 ms) and EPnP-GN (10.2 ms). The CPnP (1 ms) had less computation time than the SQPnP (2.3 ms).

### Comparing with the RANSAC algorithm

In the practice of pose estimation using PnP algorithms, outlier detection and rejection strategies such as RANSAC is often applied and only inliers are used for pose estimation, similar to the (R)EPPnP^23^ algorithm. To understand how our proposed algorithm performs comparing to the RANSAC algorithm, we self-coded the RANSAC algorithm to remove the outliers and used only inliers to estimate pose. To achieve a tradeoff between accuracy and efficiency, our RANSAC algorithm takes the Min-eig algorithm to iteratively select the consensus set, then it utilizes our proposed algorithm to estimate the pose using only the inliers.

**Figure 31:**
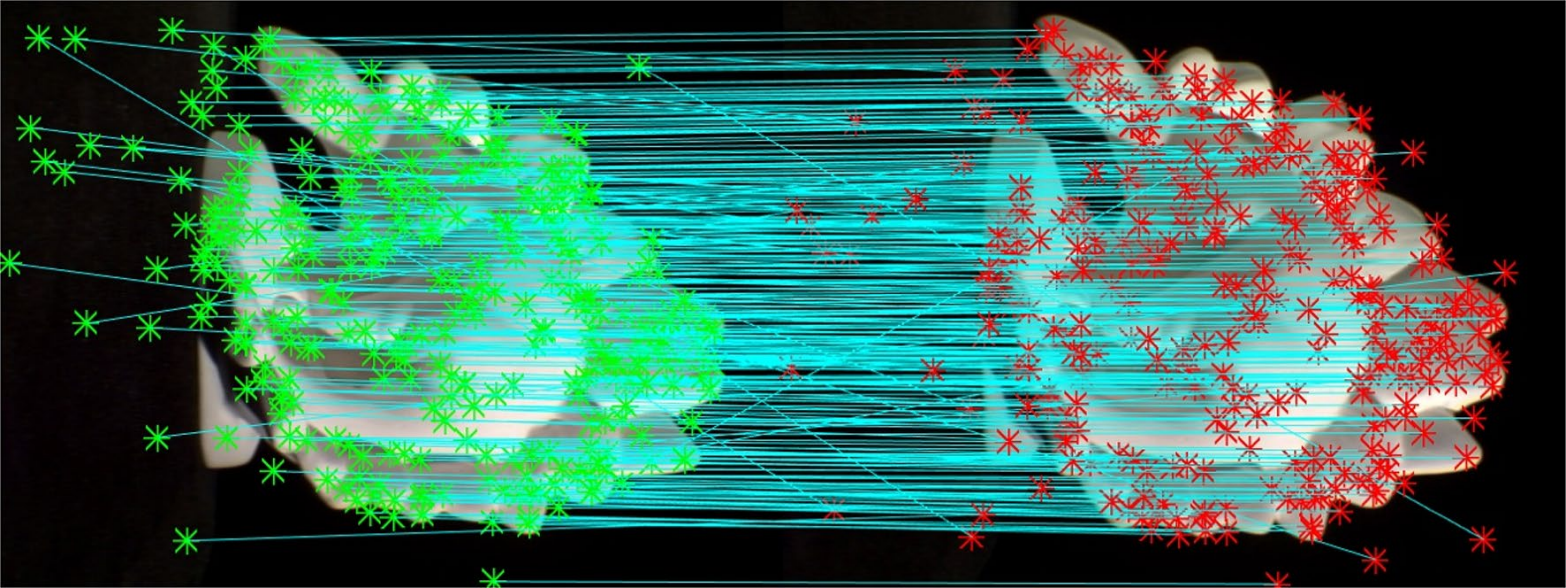
All correspondences found including inliers and outliers.

All correspondence points, including both the inliers and outliers, are obtained from the 25th and 26th image of the standard dinosaur dataset^35^ using the SIFT algorithm. Figure 31 shows all the correspondences found with straight lines connecting them. 334 correspondences were obtained in total, which more reflects real-world scenarios where hundreds of points are usually encountered.

Since the ground truth pose can be directly computed from the dataset, the rotation errors, translation errors, reprojection errors, and depth errors with and without RANSAC can be compared. Without RANSAC, the rotation error was 12.5368 degrees; the translation error was 13.0664%; the depth error from the first image was 5.0166%, and 5.0268% from the second image; the reprojection error was 0.0032 in calibrated image coordinates; the runtime was 4.1672 s (334 points).

**Figure 32:**
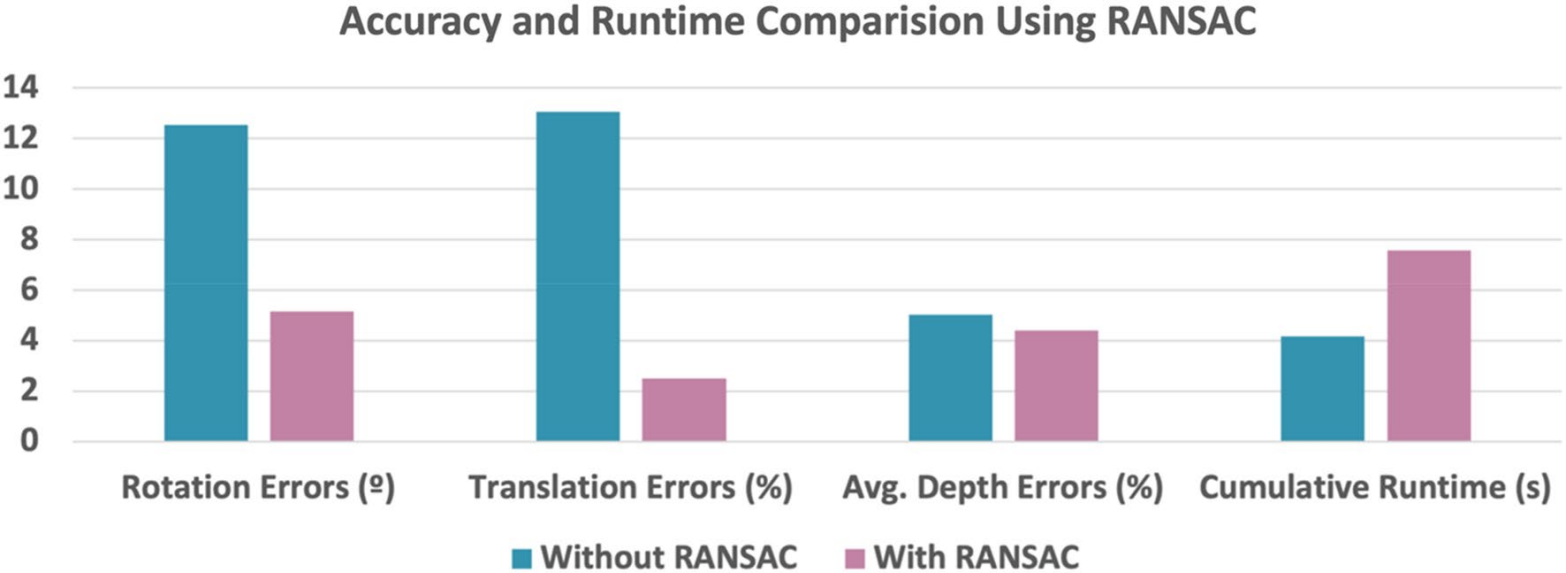
Accuracy and runtime comparison without and with RANSAC applied. With RANSAC applied before algorithm execution, the accuracy improves at the cost of increased cumulative runtime.

With RANSAC, 16 correspondence points were randomly selected for the consensus set, and 100 iterations were used to finalize such set. Therefore, the resultant consensus dataset comprising all inliers has 16 data points. The rotation error was 5.1490 degrees; the translation error was 2.5094%; the depth error from the first image was 4.3327%, and 4.4798% from the second image; the reprojection error was 0.00013 in calibrated image coordinates; the runtime was reduced to 0.0126 s due to the reduced number of points. The runtime of the RANSAC process only was 7.5512 s. Illustrative results are shown in Fig. 32, where the averaged depth error of both images, and the cumulative runtime (RANSAC and algorithm execution combined) are shown. For visualization purposes, the reprojection errors are not shown in Fig. 32 and are only numerically compared.

Using RANSAC before algorithm execution, the accuracy improves at the cost of increased cumulative runtime. The accuracy improvement is more obvious in Rotation and Translation Errors than that in Averaged Depth Errors. However, the tradeoff is the increased computation time, with most of the cost consumed in the RANSAC process.

## Discussion

In this work, we presented a positive depth-guaranteed pose estimation algorithm that estimates the camera pose with better or comparable performance to the state-of-the-art algorithms in certain aspects. The proposed Pos-dep algorithm guarantees positive depths. It solves depths with all positive entries directly within the algorithm execution process, instead of later calculating them, which ensures the positivity of depths even before the pose is estimated. With the existence of noise and outliers, our algorithm showed its robustness to various interfering conditions, while producing small reprojection errors (Figs. 3, 7, 10, 14, 17, 22, 25, 29; Tables 3, 8) and guaranteeing positive depth estimates all the time.

The way the proposed algorithm proves its tolerance on high percentage of outliers is not by detecting or removing outliers before computing the pose. In fact, without any outlier rejection scheme, the proposed algorithm demonstrates its tolerance on outliers. Of course, outliers can be screened and removed before algorithm execution, and outlier rejection scheme can be integrated as part of the algorithm. However, we are demonstrating the algorithm’s outlier tolerance capabilities itself, without extra techniques that help handle outliers, which often take place “outside” the algorithm.

Many PnP algorithms evaluate their performance on degenerate configurations such as planar or quasi-singular configurations. We studied previous works that include these configurations and found that RPnP is robust in both planar and quasi-singular point configurations, it can also deal with non-planar configurations; EPnP struggles in co-planar configurations; and LHM assumes coplanar or weak-perspective point configurations, which may limit its wide adoption. For our algorithm, we randomly generated the data points in 3D space for simulation. Thus, they are not intentionally configured to be in co-planar, nor in quasi-singular configurations. We ran simulations that had points all on the same plane parallel to the image plane, and found that our algorithm produced inconsistent results only when the points were too close to the camera. If we made sure the distance from the camera to the object was at least half the length of the data span, the algorithm always produced consistent results. From one aspect, this finding proves our algorithm’s capability of handling co-planar configuration. In real-world scenarios, points used in the rigid box case of this paper were deliberately selected to avoid degenerate configurations such as co-planar. That is, most of the points were selected to be the box corners. In addition, our algorithm was also able to handle co-planar configuration in the satellite mockup scenario. Thus, through both simulation and real-world testing, our algorithm was able to handle certain degenerate configurations.

In many of our experiments, we are aware that the number of points used starts from 10; while for many PnP algorithms, this number is valid if it is greater than 3. In addition to the family of PnP algorithms, since we are also testing the 5-pt algorithm, as well as the 8-pt algorithm, which require at least 5 or 8 points, to meet the point number requirement of all the algorithms, at least 8 points are needed. When varying the number of points, we could certainly choose the starting number to be 8. In fact, when setting the starting number to be 10 instead, it is consistent with the real-world scenarios of the study, where 10 control points were selected. Yet, in the experiment of “varying the number of points”, we chose the starting number to be 8 (8 to 30 points with an increment of 1).

Mathematically, our Pos-dep (Min-eig-Depths) algorithm finds the depth as an eigenvector of a constructed data matrix. That is, instead of directly solving for rotation matrix $R$ and translation vector ${\varvec{t}}$, it first finds all the “positive” eigenvectors (eigenvectors with all positive entries) of a data matrix, and picks the “positive eigenvector” associated with the smallest eigenvalue to be the depth vector, thus all depth values are guaranteed to be positive. To guarantee such “positive eigenvector” exists, the algorithm keeps taking randomly generated rotations as new initial guesses, until an eigenvector with all positive entries could be found. The number controlling the repetition times is a user-defined parameter. During our study, we have not encountered a case where no positive solutions could be found. However, to handle such rare case, we brute force set the resulting eigenvector with all entries equal to 1, which is a “positive” vector. Hence, the existence of such “positive” vector is proven empirically, rather than mathematically and we acknowledge that the existence of the “positive” eigenvector is achieved through empirical observation. After the “positive” eigenvector is found, the algorithm then uses Optimal Quaternion Algorithm^41–46^ to calculate a new rotation, and iteratively compares the current rotation with the new rotation under some set threshold (for example, $5^\circ$), then update ${\varvec{R}}$ if necessary (see Algorithm 1 in Methods). After the rotation is solved, the algorithm uses the solved ${\varvec{R}}$, the solved (positive) depths and the input data (observations) to calculate the translation vector ${\varvec{t}}$.

As for computational cost and efficiency, the Pos-dep/Min-eig-Depths had a runtime of less than 35 ms when the number of points was less than 30 (Fig. 20). In real-world tests, the Pos-dep reported a runtime of about 6 ms (Table 5) and 4 ms (Table 10) on the two standard datasets, and a runtime of about 10 ms on the rigid box and the satellite mockup cases. Since the time-consuming sign check and solution rejection processes have been integrated into the Pos-dep itself, the algorithm automatically handles such processes without manual and standalone checks after algorithm execution. Therefore, no runtime is wasted on the cheirality check process after the pose is solved, which has already been taken care of at the algorithm execution phase. Although such mechanism may inherently increase the runtime of the algorithm itself, it incorporates the separate sign-checking process into algorithm execution altogether, circumventing extra workloads.

There exist some limitations in terms of accuracy and runtime of the Pos-dep. As for accuracy, we do acknowledge that the proposed algorithm is less accurate than certain state-of-the-art (SOTA) algorithms as reported in Fig. 22**.** However, we found in Fig. 22a, when the point number reaches 13, the Pos-dep starts to yield a mean rotation error less than 5 degrees; in Fig. 22b, when the point number reaches 11, the median rotation error also starts to fall below 5 degrees, which is considered the threshold of good rotation estimate in this study. In Fig. 22c, when the point number reaches 13, the Pos-dep starts to yield a mean translation error less than 10%; in Fig. 22d, when the point number reaches 10, the median translation error also starts to fall below 10%, which is considered the threshold of good translation estimate in this study, and also applies to depth estimates. For reprojection errors, it can be seen from Fig. 22e and f that the Pos-dep was among the best, and when the point number reaches 14 and beyond, the Pos-dep reported an averaged reprojection error of less than 0.02 at each point number. In fact, one of the strengths of our algorithm is its ability of providing pose estimates with a small reprojection error, which is a crucial factor in pose estimation accuracy. It is also worth noting that Pos-dep performed less accurate (than EPnP) in Fig. 22, which is “varying the number of points” and is only one of the many experiments conducted in this work. In the experiment of “varying the percentage of outliers”, we can see from Fig. 14 that Pos-dep outperformed EPnP instead, on top of other benefits such as much smaller reprojection errors and positive depth estimates. It can be observed from the many experiments conducted in this paper that the Pos-dep may be less performant in one experiment, yet it can be more performant and even outperforms the most recent algorithms in another experiment (eg. Figures 15, 16, 17, 18, the Dino and the Temple datasets); and being able to constantly generate small reprojection errors with positive depth estimates, is a key advantage of the Pos-dep algorithm.

As for runtime, indeed, when the number of points $n$ = 30, the Pos-dep costs more time than other algorithms (Fig. 20). The increase on runtime with the increasing number of points is due to the increase of the dimension of the data matrix ${{\varvec{D}}}_{{\varvec{R}}}$, which is of size $2n\times 2n$. We also believe that the runtime inefficiency is not due to factors like MATLAB. The OPnP is also originally implemented in MATLAB and its runtime is around 20 ms. In our experiments, we found the runtime of OPnP on our machine, which is also implemented in MATLAB, is around 8 ms when $n$ < 30 (Fig. 20); and around 20 ms when $n$ ranges from 100 to 1000 (Fig. 21b). There is practically no significant runtime increase when using MATLAB on different machines. Therefore, we think the runtime inefficiency is due to the algorithm itself, which tries to find the eigenvector corresponding to the minimal eigenvalue from a data matrix of size $2n\times 2n$. The computation cost of eigenvector computation will then increase as $n$ goes up. We acknowledge that as $n$ increases, the Pos-dep may not be suitable for certain real-time applications, and it can be used as an offline backup solution to verify the results, particularly the results on reprojections. In the near future, we are aiming to improve the algorithm’s efficiency, possibly using parallelized programming or other multi-threaded acceleration techniques.

There also exist some limitations on the algorithms and datasets used in this study. In terms of algorithms, there exist some recent algorithms such as Uncertain-PnP or PnP(L)^26^ and QPEPs^27^. The proposed PnP(L) method integrates the feature uncertainty with “globally convergent PnP(L) solvers, leveraging a complete set of 2D and 3D uncertainties”^26^ for pose estimation. The QPEPs method studies many quadratic pose estimation problems (QPEPs) including PnP, hand–eye calibration, point-to-plane registration, etc.^27^. This work proposed a “general quaternion-based mathematical model”^27^ to unify these QPEPs. Though both algorithms have MATLAB implementations, we tried to incorporate the PnP(L) into our study, while encountered difficulties when adapting its “*method_list*” parameter and “*run_pnpl_method()*” function into our configuration. We also tried to incorporate the QPEPs method into our study. We managed to replicate some test results presented in its paper, while had trouble in interpreting the quaternions and correlating its implementations in the “*test_rel_att.m*” and “*test_stewart.m*” scripts with our configuration. As for other recent algorithms such as CPnP and SQPnP, we found their implementations were more straightforward to port into our configuration and managed to incorporate them into this paper. In the near future, we are adding more algorithms proposed recently into our comparisons to keep our work up-to-date, starting with the PnP(L) and the QPEPs that already have MATLAB implementations.

In terms of datasets, currently there exist more realistic computer vision datasets that are more suitable for real-world applications. Examples of these datasets and benchmarks include the KITTI Odometry Dataset^37^, the Robust Vision Challenge^38^, the ETH3D 2-view stereo benchmark^39^, the Heidelberg HD1K Stereo benchmark^40^, etc. For instance, the KITTI Odometry Dataset includes tasks of stereo, optical flow, visual odometry, 3D objects detection and 3D tracking. The dataset complements established datasets such as Middlebury by providing real-world benchmarks that can better reflect out-of-laboratory scenarios. Yet, in our work, we tested the algorithms using simulated data and four scenarios of real-world datasets. The first and the second scenarios are the Middlebury datasets, and the third and the fourth scenarios are self-constructed datasets. We acknowledge that to make the results more convincing, experiments with the datasets and benchmarks that better reflect real-world scenarios can be conducted. In the near future, we are expanding both the width and depth of our work by using these more realistic datasets.

Despite the limitations discussed above, we believe one of the most significant contributions of the proposed Pos-dep algorithm is its neat yet elegant mathematical derivation process. Pos-dep tries to minimize the spatial translation error as a least squares problem. It uses the mathematical fact that the minimum of the least squares problem will be solved when the sample mean is achieved. In light of the Min-eig algorithm that treats the translation as an eigenvector, we treat the depth vectors as eigenvectors. Using the knowledge of minimization with constraints, we use Lagrange multiplier to formulate an eigenvalue solution. The problem then transforms to minimizing the minimum eigenvalue of a constructed data matrix whose dimension is solely dependent on the number of correspondence points. Next, the algorithm iteratively solves depth and rotation until some threshold is met. Finally, translation is estimated using the estimated depth and rotation. Details on this mathematical derivation process can be found in Methods.

## Methods

To fully understand the technical details of our Pos-dep algorithm, we provide a complete mathematical derivation of our Pos-dep (Min-eig-Depths) algorithm from scratch, providing a mathematical foundation for the proposed algorithm. We will encounter multiple minimization problems during the following derivation process. We separate the whole process into three stages.

The first stage aims to get rid of translation ${\varvec{t}}$ from the problem, reducing the number of variables to be estimated. It starts from Eq. (3) with a rewritten form in Eq. (7). Taking ${\varvec{t}}$ as the only variable, we would be able to express ${\varvec{t}}$ using rotation and depths shown in Eq. (8). Hence, the problem onward only needs to control two variables: the rotation and the depths.

The second stage is Eq. (25), which estimates depths as the eigenvector corresponding to the minimum eigenvalue. By providing an initial guess for the rotation, the only variable becomes depth.

The final stage is Eq. (34), where the rotation is the only variable to be estimated. After the rotation and the depths are estimated, the translation is then calculated using Eq. (8) in a closed-form.

Each of these three stages tackles only one variable at a time, after executing these stages, the depth, the rotation, and the translation can be solved in a sequential manner. We will demonstrate the mathematical derivation from formulating error function to the minimization methods, the method to obtain positive depth estimates, and how to later estimate rotation and translation using these depth estimates.

### Formulating error function *J*

Assume the number of correspondence points between two images is n. Let ${{\varvec{X}}}_{1}^{{\varvec{i}}}$, ${{\varvec{X}}}_{2}^{{\varvec{i}}}$ ($i=1, 2, 3,\dots ,n$) denote the 3D position of the ith point seen from the two camera coordinate systems, respectively; let ${\varvec{R}}$ and ${\varvec{t}}$ denote the rotation and translation from the first camera coordinate system to the second. Hence, ${{\varvec{X}}}_{1}^{{\varvec{i}}}$, ${{\varvec{X}}}_{2}^{{\varvec{i}}}$ and ${\varvec{t}}$ are 3 × 1 real vectors, ${\varvec{R}}\in {\text{SO}}\left(3\right)$ is a rotation matrix. Then we have the following well-known equation for coordinate transform

1 $${{\varvec{X}}}_{2}^{{\varvec{i}}} = {\varvec{R}}{{\varvec{X}}}_{1}^{{\varvec{i}}}+{\varvec{t}}$$

Re-arranging Eq. (1) yields

2 $$0=\left({{\varvec{X}}}_{2}^{{\varvec{i}}}-{\varvec{R}}{{\varvec{X}}}_{1}^{{\varvec{i}}}\right)-{\varvec{t}}.$$

In real-world scenarios, due to the existence of noise, Eq. (2) does not equal the exact **0** vector. Therefore, we want to minimize the squared 2-norm of the right-hand side of Eq. (2) to get as close to 0 vector as possible. Since there are $n$ correspondence points and the 2-norms are all positive, we can instead minimize the sum of these squared 2-norms over all $n$ points.

3 $$\underset{{\varvec{t}}}{\mathit{min}}\sum_{i=1}^{n}{\Vert \left({{\varvec{X}}}_{2}^{{\varvec{i}}}-{\varvec{R}}{{\varvec{X}}}_{1}^{{\varvec{i}}}\right)-{\varvec{t}}\Vert }_{2}^{2},$$

where ${{\varvec{X}}}_{1}^{{\varvec{i}}}$, ${{\varvec{X}}}_{2}^{{\varvec{i}}}$ again denote the 3D positions of the $i$ th point seen from the two camera coordinate systems.

Now let ${{\varvec{x}}}_{1}^{{\varvec{i}}}$, ${{\varvec{x}}}_{2}^{{\varvec{i}}}$ (3 × 1 vectors) denote the calibrated (homogeneous) image coordinates (last entry scaled to 1) of the $i$ th point on the first and second image plane; ${\lambda }_{1}^{i}$ and ${\lambda }_{1}^{i}$ (scalars) denote the depths of the $i$ th point to the two camera’s image planes, we have the following relationships between the 3D coordinates and the calibrated image coordinates of each camera

4 $${{\varvec{X}}}_{1}^{{\varvec{i}}}={\lambda }_{1}^{i}{{\varvec{x}}}_{1}^{{\varvec{i}}}$$

5 $${{\varvec{X}}}_{2}^{{\varvec{i}}}={\lambda }_{2}^{i}{{\varvec{x}}}_{2}^{{\varvec{i}}}.$$

Then Eq. (1) can be re-written as

6 $${\lambda }_{2}^{i}{{\varvec{x}}}_{2}^{{\varvec{i}}} = {\varvec{R}}{\lambda }_{1}^{i}{{\varvec{x}}}_{1}^{{\varvec{i}}}+{\varvec{t}}.$$

Substituting ${{\varvec{X}}}_{1}^{{\varvec{i}}}$ and ${{\varvec{X}}}_{2}^{{\varvec{i}}}$ in Eq. (4) and Eq. (5) for those in Eq. (3), we have

7 $$\underset{{\varvec{t}}}{\mathit{min}}\sum_{i=1}^{n}{\Vert \left({\lambda }_{2}^{i}{{\varvec{x}}}_{2}^{{\varvec{i}}}-{\varvec{R}}{\lambda }_{1}^{i}{{\varvec{x}}}_{1}^{{\varvec{i}}}\right)-{\varvec{t}}\Vert }_{2}^{2},$$

which is a least-squares problem. The least-squares estimate of ${\varvec{t}}$ in Eq. (7) is the following sample mean ($j=1, 2, 3,\dots ,n$)

8 $${\varvec{t}}=\frac{1}{n}\sum_{j=1}^{n}{(\lambda }_{2}^{j}{{\varvec{x}}}_{2}^{{\varvec{j}}}-{\varvec{R}}{\lambda }_{1}^{j}{{\varvec{x}}}_{1}^{{\varvec{j}}}).$$

Now Substituting the expression of ${\varvec{t}}$ in Eq. (8) for that in Eq. (6), we have

9 $${\lambda }_{2}^{i}{{\varvec{x}}}_{2}^{{\varvec{i}}} = {\varvec{R}}{\lambda }_{1}^{i}{{\varvec{x}}}_{1}^{{\varvec{i}}}+\frac{1}{n}\sum_{j=1}^{n}{(\lambda }_{2}^{j}{{\varvec{x}}}_{2}^{{\varvec{j}}}-{\varvec{R}}{\lambda }_{1}^{j}{{\varvec{x}}}_{1}^{{\varvec{j}}}) .$$

Let the right-hand side minus the left-hand side of Eq. (9) and call the difference as ${e}^{i}$. Also due to noise, ${e}^{i}$ does not equal the exact zero vector $0$. The objective is to minimize the following ${e}^{i}$, or ${ne}^{i}$

10 $${e}^{i} = {\varvec{R}}{\lambda }_{1}^{i}{{\varvec{x}}}_{1}^{{\varvec{i}}}+\frac{1}{n}\sum_{j=1}^{n}{(\lambda }_{2}^{j}{{\varvec{x}}}_{2}^{{\varvec{j}}}-{\varvec{R}}{\lambda }_{1}^{j}{{\varvec{x}}}_{1}^{{\varvec{j}}})-{\lambda }_{2}^{i}{{\varvec{x}}}_{2}^{{\varvec{i}}}$$

11 $${ne}^{i} = n{\varvec{R}}{\lambda }_{1}^{i}{{\varvec{x}}}_{1}^{{\varvec{i}}}+\sum_{j=1}^{n}{(\lambda }_{2}^{j}{{\varvec{x}}}_{2}^{{\varvec{j}}}-{\varvec{R}}{\lambda }_{1}^{j}{{\varvec{x}}}_{1}^{{\varvec{j}}})-n{\lambda }_{2}^{i}{{\varvec{x}}}_{2}^{{\varvec{i}}}.$$

At this stage let’s re-format the input data ${{\varvec{x}}}_{1}^{{\varvec{i}}}$ and ${{\varvec{x}}}_{2}^{{\varvec{i}}}$ to form two new matrices ${{\varvec{\chi}}}_{1}^{{\varvec{i}}}$ and ${{\varvec{\chi}}}_{2}^{{\varvec{i}}}$

12 $${{\varvec{\chi}}}_{1}^{{\varvec{i}}}=\left[{{\varvec{x}}}_{1}^{1},\boldsymbol{ }{{\varvec{x}}}_{1}^{2},\boldsymbol{ }{{\varvec{x}}}_{1}^{3},\boldsymbol{ }\dots ,\boldsymbol{ }{{\varvec{x}}}_{1}^{{\varvec{i}}-1}, \left(1-n\right){{\varvec{x}}}_{1}^{{\varvec{i}}},\boldsymbol{ }{{\varvec{x}}}_{1}^{{\varvec{i}}+1},\boldsymbol{ }\dots ,\boldsymbol{ }{{\varvec{x}}}_{1}^{{\varvec{n}}}\right]$$

13 $${{\varvec{\chi}}}_{2}^{{\varvec{i}}}=\left[{{\varvec{x}}}_{2}^{1}, {{\varvec{x}}}_{2}^{2}, {{\varvec{x}}}_{2}^{3}, \dots , {{\varvec{x}}}_{2}^{{\varvec{i}}-1}, \left(1-n\right){{\varvec{x}}}_{2}^{{\varvec{i}}}, {{\varvec{x}}}_{2}^{{\varvec{i}}+1}, \dots , {{\varvec{x}}}_{2}^{{\varvec{n}}}\right].$$

Then, let

14 $$\overrightarrow{{\lambda }_{1}}=\left[\begin{array}{c}{\lambda }_{1}^{1}\\ {\lambda }_{1}^{2}\\ {\lambda }_{1}^{3}\\ \vdots \\ {\lambda }_{1}^{n}\end{array}\right]$$

15 $$\overrightarrow{{\lambda }_{2}}=\left[\begin{array}{c}{\lambda }_{2}^{1}\\ {\lambda }_{2}^{2}\\ {\lambda }_{2}^{3}\\ \vdots \\ {\lambda }_{2}^{n}\end{array}\right]$$

Be the two $n\times 1$ vectors containing the depths. Then Eq. (11) can be written in a much more compact form

16 $${ne}^{i} = {{\varvec{\chi}}}_{2}^{{\varvec{i}}}\overrightarrow{{\lambda }_{2}}-{\varvec{R}}{{\varvec{\chi}}}_{1}^{{\varvec{i}}}\overrightarrow{{\lambda }_{1}},$$

or in a matrix form

17 $${ne}^{i} = \left[\begin{array}{cc}-{\varvec{R}}{{\varvec{\chi}}}_{1}^{{\varvec{i}}}& {{\varvec{\chi}}}_{2}^{{\varvec{i}}}\end{array}\right]\left[\begin{array}{c}\overrightarrow{{\lambda }_{1}}\\ \overrightarrow{{\lambda }_{2}}\end{array}\right].$$

Let

18 $$\overrightarrow{\lambda }=\left[\begin{array}{c}\overrightarrow{{\lambda }_{1}}\\ \overrightarrow{{\lambda }_{2}}\end{array}\right],$$

then from Eq. (17) we have

19 $${ne}^{i} = \left[\begin{array}{cc}-{\varvec{R}}{{\varvec{\chi}}}_{1}^{{\varvec{i}}}& {{\varvec{\chi}}}_{2}^{{\varvec{i}}}\end{array}\right]\overrightarrow{\lambda }.$$

Since ${ne}^{i}$ is a 3 × 1 vector, to “minimize” ${ne}^{i}$, let’s minimize its 2-norm as the following expression

20 $${\Vert {ne}^{i}\Vert }_{2}^{2}={n}^{2}{e}^{{i}^{T}}{e}^{i}={\overrightarrow{\lambda }}^{T}\left[\begin{array}{c}-{{{\varvec{\chi}}}_{1}^{{\varvec{i}}}}^{T}{{\varvec{R}}}^{T}\\ {{{\varvec{\chi}}}_{2}^{{\varvec{i}}}}^{T}\end{array}\right]\left[\begin{array}{cc}-{\varvec{R}}{{\varvec{\chi}}}_{1}^{{\varvec{i}}}& {{\varvec{\chi}}}_{2}^{{\varvec{i}}}\end{array}\right]\overrightarrow{\lambda },$$

where ${(...)}^{T}$ denotes the transpose operation.

So far, we can introduce our error function J as

21 $$J={n}^{2}\sum_{i=1}^{n}{e}^{{i}^{T}}{e}^{i}={\overrightarrow{\lambda }}^{T}(\sum_{i=1}^{n}{M}^{i}({\varvec{R}}))\overrightarrow{\lambda },$$

where

22 $${M}^{i}\left({\varvec{R}}\right)= \left[\begin{array}{c}-{{{\varvec{\chi}}}_{1}^{{\varvec{i}}}}^{T}{{\varvec{R}}}^{T}\\ {{{\varvec{\chi}}}_{2}^{{\varvec{i}}}}^{T}\end{array}\right]\left[\begin{array}{cc}-{\varvec{R}}{{\varvec{\chi}}}_{1}^{{\varvec{i}}}& {{\varvec{\chi}}}_{2}^{{\varvec{i}}}\end{array}\right],$$

according to Eq. (20).

Next, let

23 $${{\varvec{D}}}_{{\varvec{R}}}=\sum_{i=1}^{n}{M}^{i}({\varvec{R}}).$$

Then, from Eq. (21) we have

24 $$J={\overrightarrow{\lambda }}^{T}{{\varvec{D}}}_{{\varvec{R}}}\overrightarrow{\lambda },$$

which is the formulated error function $J$. Note that ${{\varvec{D}}}_{{\varvec{R}}}$ is a function of the unknown rotation ${\varvec{R}}$ and the known image positions ${{\varvec{x}}}_{1}^{{\varvec{i}}}$ and ${{\varvec{x}}}_{2}^{{\varvec{i}}}$, where $i=1, 2, 3,\dots ,n$.

### Minimizing *J* with positive depths constraint

Now that the error function is obtained, the Min-eig algorithm searches its minimum. The Min-eig algorithm normalizes the translation vector ${\varvec{t}}$ to unit vector since there is inherent unavoidable ambiguity in the stereo image problem’s scale. Likewise, here we instead normalize the depths vector $\overrightarrow{\lambda }$ to control the scale ambiguity. The other constraint of $\overrightarrow{\lambda }$ is that all its elements must be positive (positive depths constraint).

The minimization problem can now be stated as

$$\underset{\overrightarrow{\lambda } }{\mathit{min}}{J=\overrightarrow{\lambda }}^{T}{{\varvec{D}}}_{{\varvec{R}}}\overrightarrow{\lambda }$$

*subject to*

25 $$\Vert \overrightarrow{\lambda }\Vert =1=\sqrt{{\overrightarrow{\lambda }}^{T}\overrightarrow{\lambda } }={\overrightarrow{\lambda }}^{T}\overrightarrow{\lambda }, \overrightarrow{\lambda }>0,$$

where $\overrightarrow{\lambda }>0$ indicates all elements of $\overrightarrow{\lambda }$ are positive. Such constraint has been considered in our MATLAB code, guaranteeing that all entries in the eigenvector were either all positive or all negative. If all entries were negative, we would negate them to get all-positive entries. Next, for the other constraint regarding the norm, apply Lagrange multiplier $\mu$ and Lagrange function $\mathcal{L}$:

26 $$\mathcal{L}=J+\mu (1-{\overrightarrow{\lambda }}^{T}\overrightarrow{\lambda } )$$

27 $$\frac{\partial \mathcal{L}}{\partial \overrightarrow{\lambda } }=0=2{{\varvec{D}}}_{{\varvec{R}}}\overrightarrow{\lambda }-2\mu \overrightarrow{\lambda },$$

then

28 $${{\varvec{D}}}_{{\varvec{R}}}\overrightarrow{\lambda }=\mu \overrightarrow{\lambda }.$$

Rewriting error function $J$ in Eq. (25) we can find the minimum of $J$ as

29 $${J}_{min}={\overrightarrow{\lambda }}^{T}\mu \overrightarrow{\lambda }=\mu {\overrightarrow{\lambda }}^{T}\overrightarrow{\lambda }=\mu .$$

From Eq. (28) we see that $\mu$ is an eigenvalue of ${{\varvec{D}}}_{{\varvec{R}}}$ (a $2n\times 2n$ matrix) and $\overrightarrow{\lambda }$ is the associated (unit) eigenvector. From Eq. (29) we can conclude that to minimize the error function $J$, we just need to minimize the minimum eigenvalue of ${{\varvec{D}}}_{{\varvec{R}}}$, with the associated eigenvector as the depths vector containing all positive entries. To fulfill the positive depths constraint, we need to first find all eigenvectors with all positive entries, then among them find the eigenvector associated with the smallest eigenvalue.

### Estimating rotation and translation from depths

After we find the positive depths vector $\overrightarrow{\lambda }$, we can estimate the pose using the input data and $\overrightarrow{\lambda }$. We used the Optimal Quaternion Algorithm^39–44^ (OQA, also see page 427 in Ref.^27^) to solve for rotation ${\varvec{R}}$ as follows:

Recall that Eq. (16) is

30 $${ne}^{i} = {{\varvec{\chi}}}_{2}^{{\varvec{i}}}\overrightarrow{{\lambda }_{2}}-{\varvec{R}}{{\varvec{\chi}}}_{1}^{{\varvec{i}}}\overrightarrow{{\lambda }_{1}}.$$

Let

31 $${{\varvec{D}}}_{1}={{\varvec{\chi}}}_{1}^{{\varvec{i}}}\overrightarrow{{\lambda }_{1}}$$

32 $${{\varvec{D}}}_{2}={{\varvec{\chi}}}_{2}^{{\varvec{i}}}\overrightarrow{{\lambda }_{2}},$$

be the inputs to the OQA, which are known to be dependent upon the transformed data ${{\varvec{\chi}}}_{1}^{{\varvec{i}}}$, ${{\varvec{\chi}}}_{2}^{{\varvec{i}}}$, and $\overrightarrow{\lambda }$, Eq. (30) now becomes

33 $${ne}^{i} = {{\varvec{D}}}_{2}-{\varvec{R}}{{\varvec{D}}}_{1}.$$

Recalling Eq. (21), here, we want to minimize the sum of the squared 2-norm of ${ne}^{i}$ over all $n$ points, that is (indices $i=1, 2, 3,\dots ,n$ are implicit in ${{\varvec{D}}}_{1}$ and ${{\varvec{D}}}_{2}$),

$$\underset{R}{\mathit{min}}\sum_{i=1}^{n}{\Vert {{\varvec{D}}}_{2}-{\varvec{R}}{{\varvec{D}}}_{1}\Vert }_{2}^{2}$$

*subject to*

34 $${\varvec{R}}\in SO\left(3\right).$$

Given ${{\varvec{D}}}_{1}$ and ${{\varvec{D}}}_{2}$, this problem can be analytically solved using the OQA. Recall Eq. (8), the translation ${\varvec{t}}$ is then equal to

35 $${\varvec{t}}=\frac{1}{n}\sum_{j=1}^{n}{(\lambda }_{2}^{j}{{\varvec{x}}}_{2}^{{\varvec{j}}}-{\varvec{R}}{\lambda }_{1}^{j}{{\varvec{x}}}_{1}^{{\varvec{j}}}).$$

Normalization of ${\varvec{t}}$ gives us

36 $${\varvec{t}}=\frac{{\varvec{t}}}{\Vert {\varvec{t}}\Vert }.$$

The positive depths values, are therefore also scaled by the norm of ${\varvec{t}}$

37 $$\overrightarrow{{\lambda }_{1}}=\frac{\overrightarrow{{\lambda }_{1}}}{\Vert {\varvec{t}}\Vert }$$

38 $$\overrightarrow{{\lambda }_{2}}=\frac{\overrightarrow{{\lambda }_{2}}}{\Vert {\varvec{t}}\Vert }.$$

### The iterative nature of the proposal

The rotation and depths are estimated iteratively. First, we provide an initial guess for rotation ***R*** and finds the eigenvector (depth vector) of ${{\varvec{D}}}_{{\varvec{R}}}$ with all positive or negative entries whose associated eigenvalue is the smallest; second, use OQA to solve for a new rotation estimate ***R***_***new***_. If the difference between the initial guess ***R*** and ***R***_***new***_ is within some threshold, the final estimated rotation is ***R***_***new***_; otherwise, take ***R***_***new***_ as the new initial guess and repeat the above process, until the difference between the initial guess and ***R***_***new***_ is within some threshold. We can see rotation and depths are updated iteratively.

### The Pos-dep algorithm

Based on the derivation above, we can summarize the proposed Pos-dep algorithm as follows. See Algorithm 1.

**Algorithm 1:**
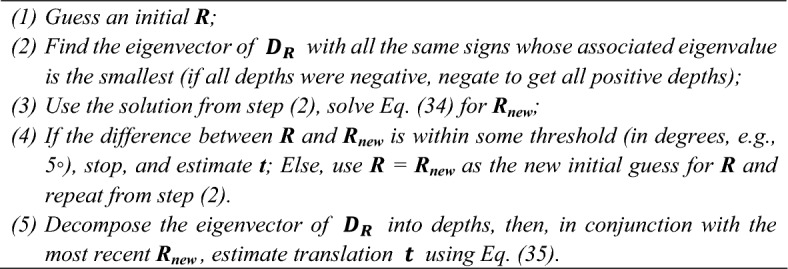
The *Pos-dep* Algorithm.

### Generating problem instances

For the simulation part, taking “varying the noise levels” as an example, we chose arbitrary calibration matrix with randomized rotation and translation; we also randomized 3D positions ${{\varvec{X}}}_{1}^{{\varvec{i}}}$ and calculated ${{\varvec{X}}}_{2}^{{\varvec{i}}}$ using Eq. (1); then we were able to generate calibrated and uncalibrated (pixel) coordinates, which are the inputs of various pose estimation algorithms. The way we added noise is as follows: we added random Gaussian noise with different noise standard deviations, known as “noise levels”, to the calibrated coordinates ${{\varvec{x}}}_{1}^{{\varvec{i}}}$, ${{\varvec{x}}}_{2}^{{\varvec{i}}}$. For each noise level, 100 tests were performed to calculate the percentage of good estimates at that noise level. In addition, the mean and median estimation errors of the algorithms were also studied.

For the real-world part, the correspondences were found using SIFT algorithm for the standard datasets, and were selected manually for the rigid box and the satellite mockup cases. The camera intrinsics in the standard datasets were available thus no calibration was needed. We self-calibrated the cameras using checkerboard patterns for the rigid box and the satellite mockup cases to obtain calibrated image coordinates. For the rigid box image and the satellite mockup image, the 3D positions of the points were manually measured using a ruler, and the 2D coordinates were obtained through MATLAB’s “getpts” control point selection, which were later converted to calibrated image coordinates using the calibration information.

### Integrating the SQPnP and CPnP algorithms

The SQPnP algorithm has been implemented in OpenCV using the *solvePnP* function with the flag option set as *SOLVEPNP_SQPNP*. We used OpenCV’s Python package “*cv2*” and ported the Python script into MATLAB using the “*py*” prefix preceding the Python expressions. In this way, Python script of SQPnP can be directly used inside MATLAB and made comparable with other algorithms.

The CPnP algorithm, like most other PnP algorithms used in this study, its MATLAB implementation is available online at: https://github.com/LIAS-CUHKSZ/CPnP-A-Consistent-PnP-Solver/tree/main/CPnP.

## Conclusion

We proposed a novel camera pose estimation algorithm, the Pos-dep/Min-eig-Depths algorithm. The algorithm was formulated based on comprehensive mathematical derivation, which solves for depth as the eigenvector associated with the minimum eigenvalue of a constructed data matrix. The algorithm was tested along with many other pose estimation algorithms under various noisy conditions in numerous simulated and real-world experiments. The proposed algorithm showed better or comparable performance than the SOTA in certain experiments, while producing small reprojection errors and guaranteeing positive depths. In the near future, we are aiming to improve the accuracy and efficiency of the proposed algorithm. We will also incorporate more recently proposed algorithms, as well as more realistic computer vision datasets into our study.

### Supplementary Information

Supplementary Information.

## Data Availability

The data that can be used to replicate the results presented in this paper is available by reasonable request after contacting the corresponding author.

## Code availability

The MATLAB code that implements the proposed algorithm and other algorithms being compared in this study, as well as the code that supports the findings of this study, is available by reasonable request after contacting the corresponding author.

## References

[1] Schönberger, J.L., Frahm, J.M. Structure-from-motion revisited. In: *Proceedings of the IEEE Conference on Computer Vision and Pattern Recognition*, pp. 4104–4113 (2016).

[2] Khairuddin AR, Talib MS, Haron H, Khairuddin AR (2015). Review on simultaneous localization and mapping (slam). 2015 IEEE International Conference on Control System, Computing and Engineering (ICCSCE).

[3] Wang Z, Menenti M (2021). Challenges and opportunities in Lidar remote sensing. Front. Remote Sens..

[4] Kim I, Martins RJ, Jang J (2021). Nanophotonics for light detection and ranging technology. Nat. Nanotechnol..

[5] Yasuda YDV, Martins LEG, Fabio AM (2021). Autonomous visual navigation for mobile robots: A systematic literature review. ACM Comput. Surv..

[6] Feng S, Yan X, Sun H (2021). Intelligent driving intelligence test for autonomous vehicles with naturalistic and adversarial environment. Nat. Commun..

[7] Xiong J, Hsiang EL, He Z (2021). Augmented reality and virtual reality displays: Emerging technologies and future perspectives. Light Sci. Appl..

[8] Dargan S, Bansal S, Kumar M (2023). Augmented reality: A comprehensive review. Arch. Computat. Methods Eng..

[9] Longuet-Higgins H (1981). A computer algorithm for reconstructing a scene from two projections. Nature.

[10] Hartley RI (1997). In defense of the eight-point algorithm. IEEE Trans. Pattern Anal. Mach. Intell..

[11] Buffington, R. L. and McInroy, J. E. Change detection for visual satellite inspection using pose estimation and image synthesis. *Proc. SPIE 8044, Sensors and Systems for Space Applications IV*, 80440A (2011).

[12] Nister D (2004). An efficient solution to the five-point relative pose problem. IEEE Trans. Pattern Anal. Mach. Intell..

[13] Five point algorithm by Sergio Agostinho. https://github.com/SergioRAgostinho/five_point_algorithm.git.

[14] F. Moreno-Noguer, V. Lepetit and P. Fua. Accurate Non-Iterative (*n*) Solution to the P*n*P Problem. *2007 IEEE 11th International Conference on Computer Vision*, Rio de Janeiro, Brazil (2007).

[15] Lepetit V, Moreno-Noguer F, Fua P (2009). EPnP: An accurate O(n) solution to the PnP problem. In. J. Comput. Vis..

[16] J. A. Hesch and S. I. Roumeliotis. A Direct Least-Squares (DLS) method for PnP. *2011 International Conference on Computer Vision*, Barcelona, Spain, pp. 383–390 (2011).

[17] Li S, Xu C, Xie M (2012). A robust O(n) solution to the perspective-n-point problem. IEEE Trans. Pattern Anal. Mach. Intell..

[18] Lu C, Hager GD, Mjolsness E (2000). Fast and globally convergent pose estimation from video images. IEEE Trans. Pattern Anal. Mach. Intell..

[19] Abdel-aziz YI, Karara HM, Hauck M (1971). Direct linear transformation from comparator coordinates into object space coordinates in close-range photogrammetry. Photogramm. Eng. Remote Sens..

[20] Zheng, Y, Kuang, Y, Sugimoto, S, Astrom, K, Okutomi, M. Revisiting the PnP Problem: A Fast, General and Optimal Solution. In: *2013 IEEE International Conference on Computer Vision* p. 2344–2351 (2013).

[21] Urban S, Leitloff J, Hinz S (2016). MLPnP—A real-time maximum likelihood solution to the perspective-n-point problem. ISPRS Ann. Photogramm. Remote Sens. Spatial Inform. Sci..

[22] MLPnP: MATLAB Toolbox by Urban, Steffen and Leitloff, Jens and Hinz, Stefan. https://github.com/urbste/MLPnP_matlab_toolbox.git.

[23] Ferraz, L, Binefa. X, Moreno-Noguer. F. Very Fast Solution to the PnP Problem with Algebraic Outlier Rejection. In: *2014 IEEE Conference on Computer Vision and Pattern Recognition*. p. 501–508 (2014).

[24] Terzakis, George, and Manolis Lourakis. A consistently fast and globally optimal solution to the perspective-n-point problem. *Computer Vision–ECCV 2020*: 16th European Conference, Glasgow, UK, August 23–28, 2020, Proceedings, Part I 16. Springer International Publishing (2020).

[25] Zeng G, Chen S, Mu B, Zeng G (2023). Cpnp: Consistent pose estimator for perspective-n-point problem with bias elimination. 2023 IEEE International Conference on Robotics and Automation (ICRA).

[26] Vakhitov A, Ferraz L, Agudo A, et al. Uncertainty-aware camera pose estimation from points and lines. *Proceedings of the IEEE/CVF Conference on Computer Vision and Pattern Recognition*. 2021: 4659–4668.

[27] Wu J, Zheng Y, Gao Z (2022). Quadratic pose estimation problems: Globally optimal solutions, solvability/observability analysis, and uncertainty description. IEEE Trans. Robot..

[28] Fischler MA, Bolles RC (1981). Random sample consensus: A paradigm for model fitting with applications to image analysis and automated cartography. Commun. ACM.

[29] Hartley R, Zisserman A (2001). Multiple View Geometry in Computer Vision.

[30] Ma et al. An Invitation to 3-D Vision: From Images to Geometric Models. New York: Springer. ISBN:978–0–387–00893–6 (2004).

[31] Torr P, Davidson C, Vernon D (2000). IMPSAC: A synthesis of importance sampling and random sample consensus to effect multi-scale image matching for small and wide baselines. European Conference on Computer Vision.

[32] Torr P, Zisserman A (2000). MLESAC: A new robust estimator with application to estimating image geometry. Comput. Vis. Image Underst..

[33] Werner T, Pajdla T. Cheirality in epipolar geometry. *Proceedings Eighth IEEE International Conference on Computer Vision*. ICCV 2001. IEEE, 2001, 1: 548–553 (2001).

[34] Li C (2021). Robust Pose Estimations Which Guarantee Positive Depths.

[35] Middlebury stereo datasets. https://vision.middlebury.edu/mview/data/.

[36] Lowe DG (2004). Distinctive image features from scale-invariant keypoints. Int. J. Comput. Vis..

[37] A. Geiger, P. Lenz and R. Urtasun, "Are we ready for autonomous driving? The KITTI vision benchmark suite," 2012 IEEE Conference on Computer Vision and Pattern Recognition, Providence, RI, USA, 2012, pp. 3354–3361, doi: 10.1109/CVPR.2012.6248074.

[38] Robust Vision Challenge: http://www.robustvision.net/index.php.

[39] T. Schöps et al. A Multi-view Stereo Benchmark with High-Resolution Images and Multi-camera Videos. 2017 *IEEE Conference on Computer Vision and Pattern Recognition* (CVPR), Honolulu, HI, USA, 2017, pp. 2538–2547.

[40] D. Kondermann, R. Nair, K. Honauer, K. Krispin, J. Andrulis, A. Brock, B. Güssefeld, M. Rahimimoghaddam, S. Hofmann, C. Brenner, and B. Jähne. The HCI Benchmark Suite: Stereo And Flow Ground Truth With Uncertainties for Urban Autonomous Driving. In *IEEE Conference on Computer Vision and Pattern Recognition Workshops* (CVPRW) (2016).

[41] Markley FL (2002). Fast quaternion attitude estimation from two vector measurements. J. Guid. Control Dyn..

[42] Markley FL, Mortari D (2000). Quaternion attitude estimation using vector observations. J. Astronaut. Sci..

[43] Mortari D (2000). Second estimator of the optimal quaternion. J. Guid. Control Dyn..

[44] Wahba G (1965). A least squares estimate of satellite attitude. SIAM Rev..

[45] Wertz, J.R. Spacecraft Attitude Determination and Control. *Springer.* Volume **73**. ISBN 978–90–277–1204–2 (1978).

[46] Jia YB (2008). Quaternions and rotations. Com. S.

