# Supplementary material for: Robust pose estimation which guarantees positive depths
Source: Sci Rep. 2023 Dec 13;13:22165. doi: 10.1038/s41598-023-49553-9 (PMC10719315; doi:10.1038/s41598-023-49553-9)
Supplement: Supplementary file 1 — Supplementary Information. [file 41598_2023_49553_MOESM1_ESM.docx]

**Supplementary Information**

(For “Robust Pose Estimation Which Guarantees Positive Depths”)

Chun Li‡^1,2,3*^ and John E. McInroy‡^1^

^1^Department of Electrical and Computer Engineering, University of Wyoming, Laramie, 82071, Wyoming, USA

^2^ Beijing Synchrotron Radiation Facility, Institute of High Energy Physics, Chinese Academy of Sciences, Beijing, 100049, China

^3^ Spallation Neutron Source Science Center, Dongguan, Guangdong, 523803, China

‡ These authors contributed equally to this work.

^*^Correspondence and requests for materials should be addressed to: C. L. ([lichun@ihep.ac.cn](mailto:lichun@ihep.ac.cn))

**Images used in the Dino dataset**

Ten pairs of Dino images were used. These images are from *dino0025.png* (inclusive) to *dino0035.png* (inclusive) in the dataset, which are shown in **Fig. S1**.


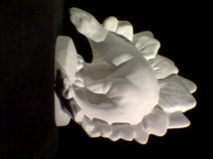

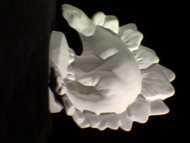

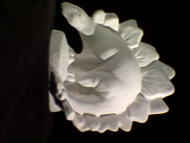

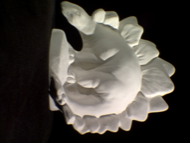

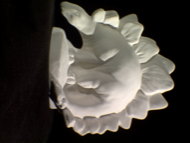


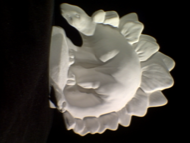

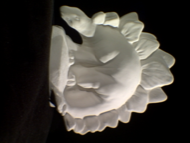

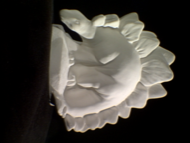

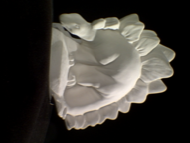

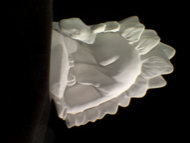

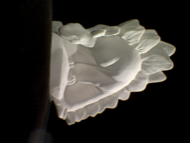


**Fig. S1**: Ten pairs of successive Dino images used in the first case of the experiments. Top: images *dino0025.png* to *dino0029.png*; Bottom: images *dino0030.png* to *dino0035.png.* Image pair 1&2 uses images *dino0025* and *dino0026*, image pair 2&3 uses images *dino0026* and *dino0027*, and so on.

**Images used in the Temple dataset**

Ten pairs of Temple images were used. These images are from *temple0040.png* (inclusive) to *temple0050.png* (inclusive) in the dataset, which are shown in **Fig. S2**.


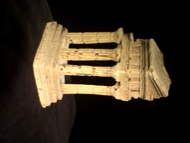

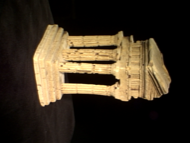

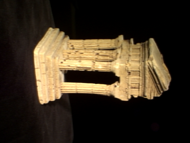

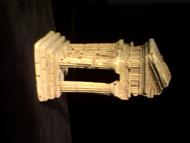

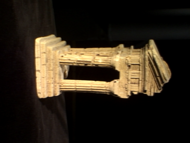


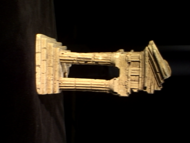

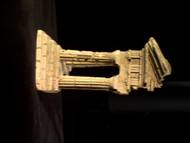

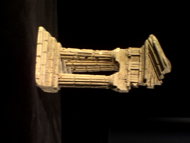

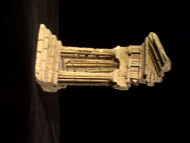

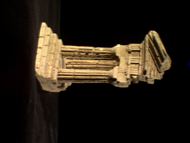

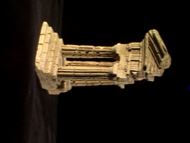


**Fig. S2**: Ten pairs of successive Temple images used in the second case of the experiments. Top: images *temple0040.png* to *temple0044.png*; Bottom: images *temple0045.png* to *temple0050.png.* Image pair 1&2 uses images *temple0040* and *temple0041*, image pair 2&3 uses images *temple0041* and *temple0042*, and so on.

**The rigid box image with control points selected**

Control points (correspondence points) on the surface of the rigid box were obtained using MATLAB’s “getpts” control point selection functionality. Points on the corners, as well as those feature-rich points on the surfaces were selected for better visualization of the 3D reconstructed model. The 3D positions of these points were manually measured using a ruler, thus human errors would be included as artificial noises, which tests the algorithms’ robustness to noise as in other real-world cases.

**
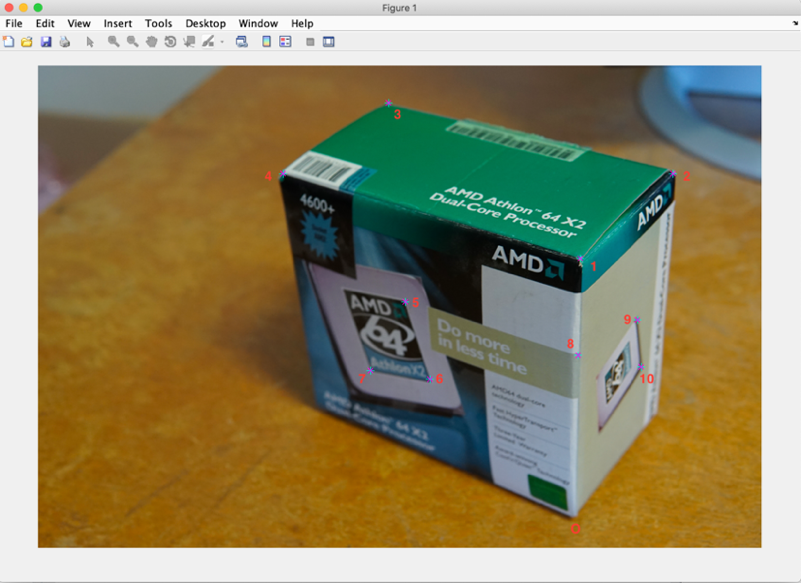
**

**Fig. S3**: Control points were selected on the rigid box image

**The transformation method from 3D-2D to 3D-3D**

The ground truth of the rigid box case (as well as the subsequent satellite mockup case) was not readily available. However, by transforming two 3D-2D problems into one 3D-3D problem, we were able to perform the study in the rigid box case and the satellite mockup case. **Fig. S4** illustrates such transformation.

Let $R_{c2}$, $R_{c1}$, $T_{c2}$ and $T_{c1}$ denote the rotation from Camera Frame to World Frame #2, the rotation from Camera Frame to World Frame #1, the translation from Camera Frame to World Frame #2, the translation from Camera Frame to World Frame #1, respectively. Let $R_{21}$ and $T_{21}$denote the pose between the two World Frames. In this problem, we do not know the ground truth of $R_{c2}$, $R_{c1}$, $T_{c2}$ and $T_{c1}$, while we do know the ground truth between the two World Frames, $R_{21}$ and $T_{21}$, which are in simple forms:

$$R_{21}=\left[ \begin{matrix} 0 & 1 & 0 \\ -1 & 0 & 0 \\ 0 & 0 & 1 \end{matrix} \right]$$

$$T_{21}=\left[ \begin{matrix} 0 \\ 1 \\ 0 \end{matrix} \right]$$

The formulas to compute $R_{21}$ and $T_{21}$ are the Rodrigues’ formula, where *^T^* denotes the transpose operation.

$$R_{21}=R_{c2}^{T}R_{c1}$$

$$T_{21}=R_{c2}^{T}(T_{c1}-T_{c2})$$

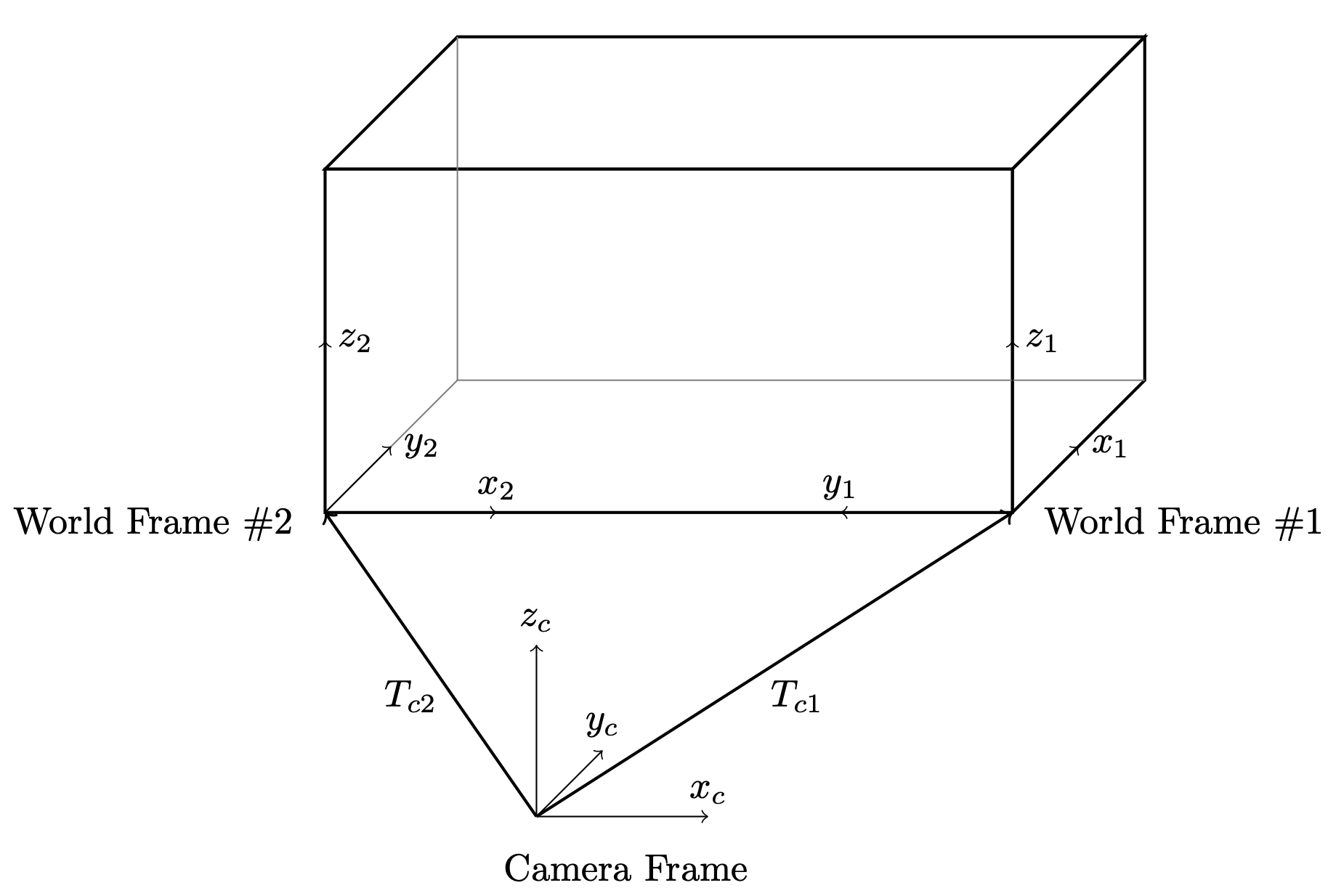


**Fig. S4**: The two world-to-camera-frame (3D-2D) pose estimations without known ground truth can be transformed into a single estimation between the two world frames (3D-3D), whose ground truth is known and in a simple form.

The first world frame was customized to be at the bottom-right corner of the box, the second being at the bottom-left corner. The dimension of the box is 145 millimeters in length, 80 millimeters in width, 135 millimeters in height. The satellite mockup case uses the similar coordinates configuration on the mockup stand. We addressed the issue of lost translation scales by using measured depths and estimated depths. $R_{c2}$, $R_{c1}$, $T_{c2}$ and $T_{c1}$ can be obtained from pose estimation algorithms, and the estimated $R_{21}$ and $T_{21}$ can be calculated and compared to the ground truth.

**The 3D reconstructed model of the rigid box**

Using the estimated pose, we can reconstruct the 3D model of the rigid box and visually compare it with the measured one, in addition to quantitative comparisons. The reconstruction is shown in **Fig. S5**. We can evaluate the estimation accuracy by visually inspecting how close the reconstructed model matches the measured model.


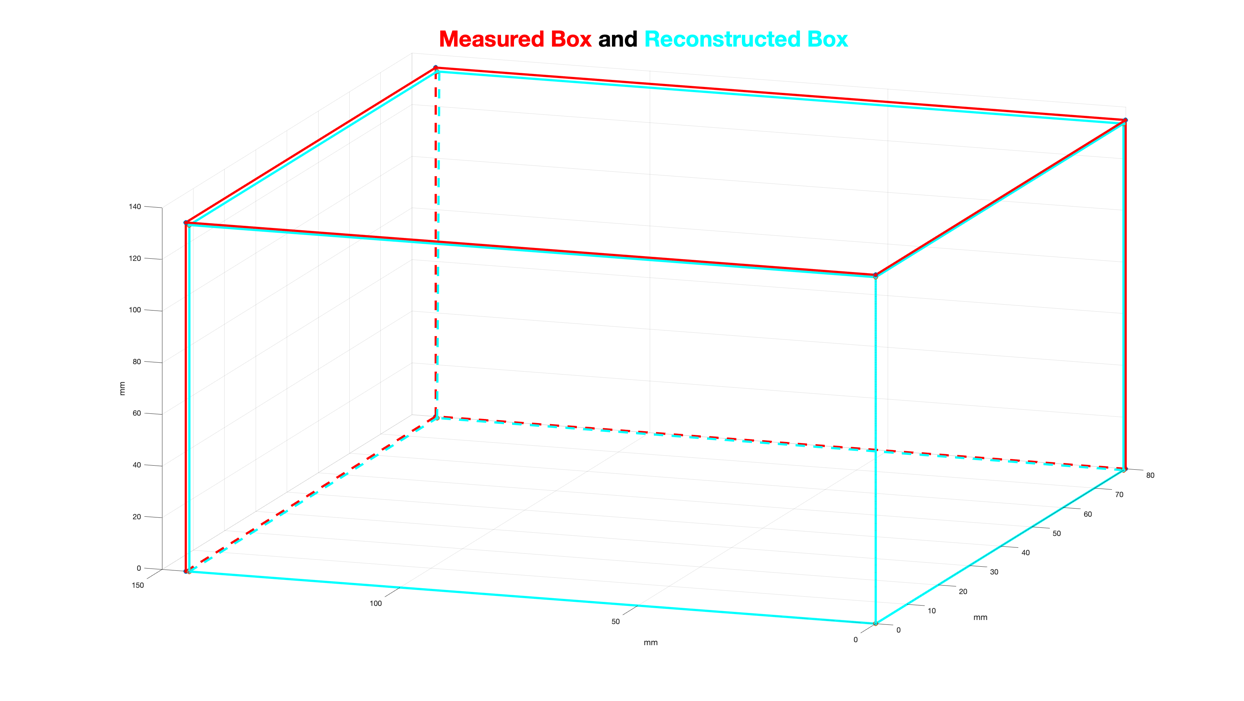


**Fig. S5**: 3D reconstruction using our Pos-dep estimates. The dimensions of the box were estimated using the Pos-dep pose estimates. Red: measured. Cyan: reconstructed.

**The satellite mockup image with correspondences selected**

Ten correspondences were selected and labeled on the mockup. The configuration of the two coordinate systems is also shown in **Fig. S6**. The similar 3D-2D to 3D-3D transform method used in the rigid box case was applied in the satellite mockup case.

We can see some of the selected points were almost in coplanar configuration (we did not intentionally choose such configuration and we were not meant to specifically test the algorithm’s capability of handling degenerate cases), which is problematic for many PnP algorithms. However, our Pos-dep can still handle such degenerate case well with good pose estimates, see “Results on the satellite mockup image” section in the main text. The 3D positions of these points were measured using the same ruler used in the rigid box case, and human errors would certainly be included as artificial noises, which tests the algorithms’ robustness to noise as in other real-world cases.

**
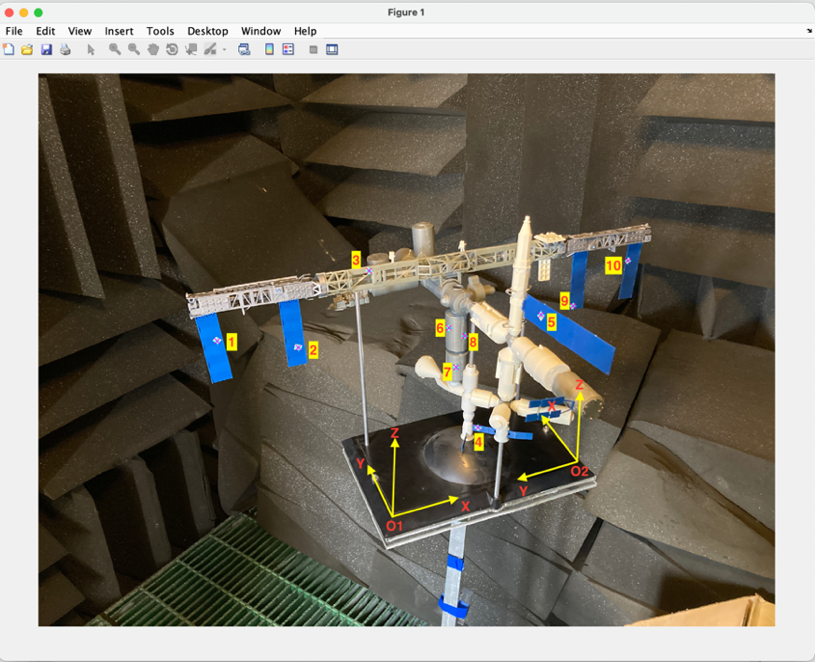
**

**Fig. S6**: Correspondence points were selected for the satellite mockup case. The two World Frames whose ground truth relative pose was readily available are also shown.
